# Interleukin-6 and YKL-40 predicted recurrent stroke after ischemic stroke or TIA: analysis of 6 inflammation biomarkers in a prospective cohort study

**DOI:** 10.1186/s12974-022-02467-1

**Published:** 2022-06-06

**Authors:** Jiejie Li, Jinxi Lin, Yuesong Pan, Mengxing Wang, Xia Meng, Hao Li, Yilong Wang, Xingquan Zhao, Haiqiang Qin, Liping Liu, Yongjun Wang, the CNSR-III Investigators

**Affiliations:** 1 Department of Neurology, Beijing Tiantan Hospital, Capital Medical University, No.119 Road Nansihuanxi, Fengtai District, Beijing, 100075 China; 2 China National Clinical Research Center for Neurological Diseases, Beijing, China; 3 Research Unit of Artificial Intelligence in Cerebrovascular Disease, Chinese Academy of Medical Sciences, Beijing, 2019RU018 China; 4 Center for Excellence in Brain Science and Intelligence Technology, Chinese Academy of Sciences, Beijing, China; 5 Advanced Innovation Center for Human Brain Protection, Capital Medical University, Beijing, China

**Keywords:** Cerebrovascular disease, Inflammation, Biomarker, Recurrence, Disability, Mortality

## Abstract

**Objective:**

Contribution of individual and combined inflammatory markers in prognosis after stroke was still undefined. We aimed to investigate the association of systemic and local vascular inflammatory markers and recurrent stroke as well as impact on poor functional outcome.

**Methods:**

In this pre-specified substudy of the Third China National Stroke Registry (CNSR-III), 10,472 consecutive acute ischemic stroke or TIA patients with available centralized-measured levels of Interleukin-6 (IL-6), high sensitive C-reactive protein (hsCRP), IL-1 receptor antagonist (IL-1Ra), lipoprotein-associated phospholipase A_2_ mass (Lp-PLA_2_) and activity (Lp-PLA_2_-A), and YKL-40 from 171 sites were enrolled. The primary outcomes consisted of stroke recurrence and poor functional outcome defined as modified Rankin Scale (mRS) score of 2–6 within 1 year.

**Results:**

There were 1026 (9.8%) and 2395 (23.4%) patients with recurrent stroke and poor functional outcome within 1 year. The highest quartiles of IL-6 (adjusted HR, 1.36; 95% CI 1.13–1.64; *P* = 0.001), hsCRP (adjusted HR, 1.41; 95% CI 1.17–1.69; *P* = 0.0003) and YKL-40 (adjusted HR, 1.28; 95% CI 1.06–1.56; *P* = 0.01) were associated with increased risk of recurrent stroke; and the highest quartiles of IL-6 (adjusted OR 1.93; 95% CI 1.64–2.27; *P* < 0.0001), IL-1Ra (adjusted OR 1.60; 95% CI 1.37–1.87; *P* < 0.0001), hsCRP (adjusted OR 1.60; 95% CI 1.37–1.86; *P* < 0.0001) and YKL-40 (adjusted OR 1.21; 95% CI 1.03–1.42; *P* = 0.02) were correlated with increased risk of poor functional outcome. In the multivariate stepwise regression analysis including all markers with backward selection, elevated levels of IL-6 or YKL-40 were associated with recurrent stroke (IL6: OR, 1.34; 95% CI 1.19–1.52; *P* < 0.0001; YKL-40: OR, 1.01; 95% CI 1.01–1.03; *P* = 0.004) and poor functional outcome (IL6: OR, 1.68; 95% CI 1.46–1.93; *P* < 0.0001; YKL-40: OR, 1.02; 95% CI 1.01–1.03; *P* = 0.0001). Adding IL-6 and YKL-40 significantly increased the area under the receiver operating characteristic curves for the prediction models of Essen Stroke Risk Score (0.03, *P* < 0.0001) and Totaled Health Risks in Vascular Events Score (0.07, *P* < 0.0001), and yielded continuous net reclassification improvement (19.0%, *P* < 0.0001; 33.0, *P* < 0.0001).

**Conclusions:**

In the patients with ischemic stroke or TIA, IL-6 and YKL-40 were independently associated with recurrent stroke and poor functional outcome, and improved risk classification of clinical risk algorithms.

**Supplementary Information:**

The online version contains supplementary material available at 10.1186/s12974-022-02467-1.

## Background

Despite widespread use of existing secondary prevention therapy, substantial residual risk of recurrent ischemic events after stroke persisted, varying from approximately 25–30% at 5 years [1]. Traditional risk factors do not explain all epidemiologic features of ischemic vascular event, and there is an urgent need for unraveling new therapeutic targets to reduce the residual risk [2].

Inflammation has been pinpointed as a key regulatory process that links multiple risk factors for ischemic stroke. Some previous epidemiology studies showed that the markers for systemic inflammation, such as interleukin-6 (IL-6), [3] IL-1 receptor antagonist (IL-1Ra) [4, 5] and high sensitive C-reactive protein (hsCRP), [6] and local vascular inflammation produced in atherosclerotic lesions, such as lipoprotein-associated phospholipase A_2_ mass (Lp-PLA_2_) and activity (Lp-PLA_2_-A) [7] and YKL-40 [8, 9] were associated with first or subsequent stroke or functional outcome. This revolution in the thinking about the pathophysiology of atherosclerosis has begun to provide clinical insight and aid patient management, which has been verified in recent randomized trials showing the cardiovascular benefits with anti-inflammatory treatment targeting IL-1 to IL-6 to hsCRP pathways in the patients with coronary disease [10–12].

However, questions remained, such as the role of IL-1Ra and YKL-40 in recurrent stroke was unclear and controversy still existed regarding the prognostic role of hsCRP, Lp-PLA_2_ and Lp-PLA_2_-A in recurrent stroke [13–16]. Moreover, current guidelines do not yet recommend routine measurement of inflammatory markers for risk prediction after stroke, and there is limited data regarding the contribution of combined atherosclerotic inflammatory markers in recurrence after stroke [16, 17].

The Third China National Stroke Registry (CNSR-III) included patients with acute ischemic stroke and transient ischemic attack (TIA) aiming to identify the biological markers for the prognosis and facilitate early evaluation and identification of patients at high risk [18]. In particular, we sought to investigate the contribution of individual and combined atherosclerotic inflammatory markers in recurrent stroke and functional outcome in this biomarker substudy of the CNSR-III.

## Methods

### Study design and participants

The design of the CNSR-III has been described in detail previously [18]. In brief, the CNSR-III was a nationwide prospective registry for patients with acute ischemic stroke and TIA presented to hospitals between August 2015 and March 2018 in China. A total of 201 study sites were included from 22 provinces and 4 municipalities. In the pre-specified biomarker substudy of the CNSR-III, fasting blood samples were collected within 24 h of admission from 171 (85%) voluntary study sites. Participants were consecutively recruited if meeting the following criteria: (1) age > 18 years; (2) diagnosis within 7 days of ischemic stroke and TIA; (3) informed consent from participant or legally authorized representative. The CNSR-III was approved by ethics committee at Beijing Tiantan Hospital and all participating centers.

### Baseline data collection

Trained research coordinators at each site collected information of age, prestroke modified Rankin Scale (mRS) score and National Institutes of Health Stroke Scale (NIHSS) score through a direct interview at admission, and baseline data from medical records, including sex, body mass index (BMI, the weight in kilograms divided by the square of the height in meters), cigarette smoking, medical history of hypertension, diabetes, hypercholesterolemia, ischemic stroke and coronary heart disease, and laboratory test of leukocyte counts. The TOAST (Trial of ORG 10,172 in Acute Stroke Treatment) criteria were used for centralized etiology classification of ischemic stroke [19, 20].

### Sample collection and measurements of markers

The median time of sampling was 55 h (interquartile range: 27–96 h) after index event onset. Specimens were extracted, aliquoted and transported through cold chain to the core laboratory in Beijing Tiantan Hospital. All specimens were stored at − 80 °C until assays were performed centrally and blindly. The concentrations of IL-6, IL-1Ra, Lp-PLA_2_ and YKL-40 were determined by using enzyme-linked immunosorbent assay kits (catalogue number: PHS600C for IL-6, PDRA00B for IL-1Ra, DPLG70 for Lp-PLA_2_ and DC3L10 for YKL-40, R&D Systems, Inc, Minneapolis, MN, USA). Lp-PLA_2_-A was measured with an automatic enzyme assay system on a Hitachi 7600 analyzer (PLAC test for Lp-PLA_2_-A, Diazyme Laboratories, Inc., Poway, CA), and hsCRP and LDL-C were detected on Roche Cobas C501 and C702 analyzers, respectively.

### Follow-up and outcomes

The detailed follow-up procedure of the CNSR-III has been previously described [18]. In brief, participants were followed up by face-to-face or telephone interview at 3 months and 1–5 year annually by trained research personnel, collecting information of any death, stroke recurrence and cardiovascular events, and assessing patient’s modified Rankin Scale (mRS) score ranging from 0 (no symptoms) to 6 (death) during the follow-up period. Confirmation of stroke was sought from the treating hospital, and suspected recurrent stroke without hospitalization was judged by independent endpoint judgment committee. Fatality was either confirmed on a death certificate from the attended hospital or the local citizen registry.

The primary outcomes consisted of stroke recurrence (ischemic or hemorrhagic) and poor functional outcome defined as an mRS score of 2–6 within 1 year. Outcome of dependence or death defined as an mRS score of 3–6 within 1 year was also assessed in the sensitivity analysis. The secondary outcomes included composite vascular events (ischemic stroke, hemorrhagic stroke, myocardial infarction, or cardiovascular death).

### Statistical analysis

Distribution of all markers were skewed based on Kolmogorov–Smirnov tests. Categorical variables were presented as percentages and continuous variables as medians with interquartile ranges. Baseline characteristics were analyzed by Chi-square statistics for the categorical variables and Kruskal–Wallis test for the skewed continuous and ordinal variables.

Each marker was assessed as continuous variable after logarithm transformation or according to quartiles as follows: IL-6: quartile 1 (Q1): ≤ 1.6 ng/L; quartile 2 (Q2): 1.6–2.6 ng/L; quartile 3 (Q3): 2.6–5.1 ng/L; quartile 4 (Q4): > 5.1 ng/L; IL-1Ra: Q1: ≤ 254.6 ng/L; Q2: 254.6–341.3 ng/L; Q3: 341.3–497.8 ng/L; Q4: > 497.8 ng/L; hsCRP: Q1: ≤ 0.8 mg/L; Q2: 0.8–1.8 mg/L; Q3: 1.8–4.7 mg/L; Q4: > 4.7 mg/L; Lp-PLA_2_: Q1: ≤ 127.6 μg/L; Q2: 127.6 to 175.0 μg/L; Q3: 175.0–225.1 μg/L; Q4: > 225.1 μg/L; Lp-PLA_2_-A: Q1: ≤ 128.0 nmol/min/ml; Q2: 128.0 to 162.2 nmol/min/ml; Q3: 162.2 to 194.6 nmol/min/ml; Q4: > 194.6 nmol/min/ml; YKL-40: Q1: ≤ 38.1 mg/L; Q2: 38.1–65.3 mg/L, Q3: 65.3–123.3 mg/L, Q4: > 123.3 mg/L. Patients were classified into 4 groups according to medians of IL-6 and YKL-40 when analyzing the effect of combined markers on outcomes.

We evaluated the associations between outcomes and individual or combined markers with the use of crude and multivariate Cox proportional hazards models or logistic regression models. The Kaplan–Meier survival curves were applied to depict the occurrence of recurrent stroke and analyzed using the log-rank univariate tests. We further evaluated the pattern and magnitude of correlation between each marker on a continuous scale and risk of stroke recurrence using multivariable Cox regression models with restricted cubic splines. Ordinal logistic regression was also applied to estimate the common odds ratio for a shift in the direction of a worse outcome on the mRS score according to marker levels. Taking into account the interactive nature of explanatory inflammatory markers, multivariate stepwise logistic regression analysis was further used to analyze the data until the model contained only significant terms. The potential confounders were demographic factors, prior published traditional or clinical risk factors, index event and medications used during follow-up period. The unadjusted and adjusted hazard ratios (HRs), Odd ratios (ORs) or common ORs and their 95% confidence intervals (CIs) were calculated.

The receiver-operator curve (ROC) with area under curve was used. In ischemic stroke patients, Essen Stroke Risk Score [21, 22] was developed to identify patients at highest risk of subsequent vascular event and the Totaled Health Risks in Vascular Events Score [23] was useful to predict the functional outcome and mortality. We therefore calculate the c-statistics and net reclassification index to evaluate improvement in risk classification by inflammatory markers over these clinical risk scores.

A 2-sided *P* value of < 0.05 was considered to indicate statistical significance. SAS software, version 9.4 (SAS Institute, Inc, Cary, NC) was used for all statistical analyses. Data were analyzed on April, 2020.

## Result

### Patient characteristics

A total of 10,472 consecutive patients with available levels of inflammatory markers participated in this substudy of the CNSR-III (Additional file 1: Fig. S1), and 255 patients were lost within 1 year (follow-up rate 97.6%). The baseline characteristics were well balanced between patients included and those not included, except that the patients enrolled were more likely to have prior ischemic stroke, diabetes, hypercholesterolemia and atrial fibrillation (Additional file 1: Table S1). The baseline characteristics mostly differed according to the marker concentrations (Table 1 and Additional file 1: Tables S2–S8). Specifically, patients with elevated levels of both IL-6 and YKL-40 were more likely older, female and non-smokers, and had lower BMI, higher NIHSS and mRS scores, index event of ischemic stroke, higher leukocyte count, and more likely had histories of ischemic stroke, diabetes, hypertension, coronary heart disease and atrial fibrillation, but no hypercholesterolemia (Table 1). Median marker levels by participants’ characteristics are provided in Additional file 1: Table S1.

**Table 1:** Distribution of baseline characteristics according to levels of IL-6 and YKL-40

	Group 1*	Group 2*	Group 3*	Group 4*	*P* value
Age (year), median (IQR)	57 (50–64)	64 (58–71)	60 (53–67)	69 (61–76)	< 0.0001
Male, no. (%)	2355 (73.7)	1268 (62.1)	1511 (74.0)	2031 (63.6)	< 0.0001
Body mass index, median (IQR)	24.8 (22.9–26.7)	24.2 (22.4–26.3)	24.7 (22.9–26.8)	24.2 (22.3–26.3)	< 0.0001
Smoking, no. (%)	1534 (48.0)	779 (38.2)	1071 (52.5)	1270 (39.8)	< 0.0001
Medical history, no. (%)					
Ischemic stroke	563 (17.6)	422 (20.7)	422 (20.7)	830 (26.0)	< 0.0001
Diabetes	725 (22.7)	460 (22.5)	519 (25.4)	806 (25.2)	0.02
Hypertension	1893 (59.3)	1318 (64.6)	1279 (62.7)	2096 (65.6)	< 0.0001
Hypercholesterolemia	284 (8.9)	135 (6.6)	174 (8.5)	276 (8.6)	0.02
Coronary heart disease	258 (8.1)	189 (9.3)	202 (9.9)	486 (15.2)	< 0.0001
Atrial fibrillation	97 (3.0)	89 (4.4)	137 (6.7)	430 (13.5)	< 0.0001
Index event, no. (%)					
Ischemic stroke	2896 (90.6)	1872 (91.7)	1934 (94.8)	3051 (95.5)	< 0.0001
TIA	299 (9.4)	169 (8.3)	107 (5.2)	144 (4.5)	< 0.0001
Baseline NIHSS, median (IQR)	3 (1–5)	3 (1–5)	4 (2–7)	4 (2–7)	< 0.0001
mRS score before the onset of index events ≥ 2, no. (%)	212 (6.6)	161 (7.9)	185 (9.1)	362 (11.3)	< 0.0001
Leukocyte count (× 10^9^/L), median (IQR)	6.7 (5.6–8.0)	6.5 (5.4–8.0)	7.3 (6.1–8.9)	7.2 (5.9–8.8)	< 0.0001

*IL-6* interleukin-6, *IQR* interquartile range, *NIHSS* National Institutes of Health Stroke Scale, *mRS* modified Rankin Scale, *LDL-C* low-density lipoprotein cholesterol

*Patients were classified into 4 groups according to medians of IL-6 (2.6 ng/L) and YKL-40 (65.3 mg/L). Group 1: IL-6 ≤ 2.6 ng/L and YKL-40 ≤ 65.3 mg/L; group2: IL-6 ≤ 2.6 ng/L and YKL-40 > 65.3 mg/L; group 3: IL-6 > 2.6 ng/L and YKL-40 ≤ 65.3 mg/L; group 4: IL-6 > 2.6 ng/L and YKL-40 > 65.3 mg/L

Spearman correlation analyses showed that none of the biomarker levels were strongly correlated (defined as a Spearman correlation coefficient *r*^2^ ≥ 0.5) with any other biomarker, except Lp-PLA_2_ and Lp-PLA_2_-A (*r*^2^ = 0.74, *P* < 0.0001; Additional file 1: Table S10). IL-6 and hsCRP were moderately correlated (*r*^2^ = 0.45, *P* < 0.0001), reflecting similar biological pathways, while the others were mildly correlated (*r*^2^ < 0.3; Additional file 1: Table S9).

### Markers and recurrent vascular events

There were 1026 (9.8%) patients with recurrent stroke within 1 year. After adjustment for potential confounders, the highest quartiles of IL-6 (adjusted HR 1.36; 95% CI 1.13–1.64; *P* = 0.001), hsCRP (adjusted HR 1.41; 95% CI 1.17–1.69; *P* = 0.0003) and YKL-40 (adjusted HR 1.28; 95% CI 1.06–1.56; *P* = 0.01) were associated with increased risk of recurrent stroke (Table 2 and Additional file 1: Fig. S2). Similar results were observed when analyzed in the continuous model (Table 2). In the Cox regression model with restricted cubic spline, these associations persisted (Additional file 1: Fig. S3). As for the outcome of composite vascular event, the highest quartiles of IL-6, hsCRP and YKL-40 were, respectively, correlated with 39%, 45% and 32% increased risk after adjustment (Additional file 1: Table S10). In the multivariate stepwise regression analysis with backward selection, the associations between IL-6 (OR 1.34; 95% CI 1.19–1.52; *P* < 0.0001) or YKL-40 (OR 1.01; 95% CI 1.01–1.03; *P* = 0.004) and recurrent stroke remained significant (Additional file 1: Fig. S4). ROC analysis indicated that the optimal cut-off values for IL-6 and YKL-40 were 2.77 ng/L and 75.64 mg/L, respectively.

**Table 2:** Associations of individual inflammation marker or combination of IL-6 and YKL-40 with recurrent stroke within 1 year

Marker and levels *		Events [no., (%)]	Model 1^†^		Model 2^‡^		Model 3^§^		Model 4^||^	
			HR (95% CI)	*P* value	HR (95% CI)	*P* value	HR (95% CI)	*P* value	HR (95% CI)	*P* value
IL-6 (ng/L)	Q1	213 (8.1)	Reference	–	Reference	–	Reference	–	Reference	–
	Q2	212 (8.1)	1.00 (0.83–1.21)	0.99	0.92 (0.76–1.11)	0.38	0.91 (0.75–1.11)	0.34	0.90 (0.74–1.10)	0.30
	Q3	262 (10.0)	1.25 (1.04–1.49)	0.02	1.07 (0.89–1.29)	0.47	1.10 (0.90–1.32)	0.37	1.08 (0.89–1.30)	0.46
	Q4	339 (13.0)	1.69 (1.42–2.00)	< 0.0001	1.35 (1.12–1.62)	0.002	1.37 (1.14–1.66)	0.001	1.36 (1.13–1.64)	0.001
	Continuous model		1.32 (1.23–1.41)	< 0.0001	1.21 (1.12–1.30)	< 0.0001	1.21 (1.12–1.31)	< 0.0001	1.21 (1.12–1.31)	< 0.0001
IL-1Ra (ng/L)	Q1	238 (9.1)	Reference	–	Reference	–	Reference	–	Reference	–
	Q2	249 (9.5)	1.05 (0.88–1.26)	0.57	1.01 (0.85–1.21)	0.91	1.02 (0.85–1.22)	0.84	1.02 (0.85–1.22)	0.84
	Q3	282 (10.8)	1.21 (1.02–1.43)	0.03	1.10 (0.92–1.31)	0.31	1.10 (0.92–1.31)	0.32	1.09 (0.91–1.31)	0.34
	Q4	257 (9.8)	1.11 (0.93–1.32)	0.25	0.98 (0.82–1.18)	0.85	1.00 (0.83–1.21)	0.98	0.98 (0.81–1.19)	0.85
	Continuous model		1.09 (0.99–1.20)	0.09	1.03 (0.93–1.14)	0.60	1.03 (0.93–1.15)	0.53	1.03 (0.92–1.14)	0.63
hsCRP (mg/L)	Q1	212 (8.1)	Reference	–	Reference	–	Reference	–	Reference	–
	Q2	218 (8.4)	1.05 (0.87–1.27)	0.63	1.03 (0.85–1.24)	0.79	1.03 (0.85–1.26)	0.74	1.03 (0.85–1.25)	0.77
	Q3	264 (10.1)	1.28 (1.07–1.53)	0.007	1.20 (1.00–1.44)	0.05	1.20 (0.99–1.44)	0.07	1.18 (0.98–1.42)	0.09
	Q4	332 (12.7)	1.66 (1.40–1.98)	< 0.0001	1.42 (1.19–1.70)	0.0001	1.42 (1.18–1.71)	0.0002	1.41 (1.17–1.69)	0.0003
	Continuous model		1.15 (1.11–1.20)	< 0.0001	1.10 (1.05–1.15)	< 0.0001	1.10 (1.05–1.16)	< 0.0001	1.10 (1.05–1.15)	< 0.0001
Lp-PLA_2_ (μg/L)	Q1	242 (9.2)	Reference	–	Reference	–	Reference	–	Reference	–
	Q2	237 (9.1)	0.99 (0.83–1.18)	0.90	1.01 (0.84–1.21)	0.91	1.00 (0.83–1.20)	0.99	1.00 (0.83–1.19)	0.96
	Q3	267 (10.2)	1.12 (0.94–1.33)	0.21	1.14 (0.96–1.36)	0.13	1.06 (0.88–1.27)	0.53	1.05 (0.88–1.26)	0.59
	Q4	280 (10.7)	1.18 (0.99–1.40)	0.06	1.17 (0.98–1.39)	0.08	1.09 (0.90–1.32)	0.37	1.08 (0.89–1.31)	0.42
	Continuous model		1.17 (1.02–1.35)	0.03	1.17 (1.01–1.35)	0.03	1.10 (0.94–1.28)	0.25	1.09 (0.93–1.27)	0.30
Lp-PLA_2_-A (nmol/min/ml)	Q1	230 (8.8)	Reference	–	Reference	–	Reference	–	Reference	–
	Q2	233 (8.9)	1.02 (0.85–1.22)	0.85	1.02 (0.85–1.22)	0.84	1.00 (0.83–1.20)	0.96	0.99 (0.82–1.19)	0.91
	Q3	282 (10.8)	1.25 (1.05–1.48)	0.01	1.24 (1.04–1.47)	0.02	1.18 (0.98–1.41)	0.08	1.17 (0.97–1.40)	0.09
	Q4	281 (10.8)	1.25 (1.05–1.49)	0.01	1.26 (1.05–1.50)	0.01	1.18 (0.98–1.43)	0.09	1.17 (0.97–1.42)	0.10
	Continuous model		1.30 (1.08–1.56)	0.006	1.30 (1.08–1.57)	0.006	1.20 (0.98–1.47)	0.07	1.19 (0.97–1.46)	0.09
YKL-40 (mg/L)	Q1	217 (8.3)	Reference	–	Reference	–	Reference	–	Reference	–
	Q2	238 (9.1)	1.10 (0.92–1.32)	0.31	1.05 (0.87–1.27)	0.59	1.06 (0.88–1.29)	0.53	1.05 (0.87–1.28)	0.60
	Q3	253 (9.7)	1.18 (0.98–1.41)	0.08	1.05 (0.87–1.27)	0.64	1.07 (0.88–1.29)	0.52	1.05 (0.87–1.28)	0.59
	Q4	318 (12.2)	1.52 (1.28–1.81)	< 0.0001	1.26 (1.05–1.53)	0.02	1.30 (1.07–1.58)	0.008	1.28 (1.06–1.56)	0.01
	Continuous model		1.24 (1.15–1.35)	< 0.0001	1.13 (1.03–1.24)	0.01	1.15 (1.05–1.26)	0.004	1.14 (1.04–1.25)	0.006
IL-6 + YKL-40	Q1	248 (7.8)	Reference	–	Reference	–	Reference	–	Reference	–
	Q2	177 (8.7)	1.13 (0.93–1.37)	0.23	1.05 (0.86–1.28)	0.66	1.08 (0.88–1.32)	0.48	1.07 (0.87–1.31)	0.51
	Q3	207 (10.1)	1.34 (1.11–1.61)	0.002	1.21 (1.00–1.45)	0.05	1.24 (1.03–1.51)	0.03	1.24 (1.02–1.50)	0.03
	Q4	394 (12.3)	1.66 (1.42–1.95)	< 0.0001	1.34 (1.12–1.59)	0.001	1.38 (1.16–1.65)	0.0004	1.37 (1.14–1.63)	0.0007

*HR* hazard ratio, *CI* confidence intervals, *Q1* quartile 1, *Q2* quartile 2, *Q3* quartile 3, *Q4* quartile 4, *IL-6* interleukin-6, *IL-1Ra* interleukin-1 receptor antagonist, *hsCRP* high-sensitive C-reactive protein, *Lp-PLA*_*2*_ lipoprotein-associated phospholipase A_2_, *Lp-PLA*_*2*_*-A* lipoprotein-associated phospholipase A_2_ activity

*All markers were categorized into 4 even groups by quartiles. In the continuous model, the hazard ratios correspond to per-unit increment of logarithm of marker value

^†^Model 1: unadjusted

^‡^Model 2: adjusted for age, sex, body mass index, smoking, index event, medical histories of atrial fibrillation, coronary heart disease, ischemic stroke, diabetes, hypertension and hypercholesterolemia, baseline NIHSS score and baseline leukocyte count

^§^Model 3: adjusted for all factors in model 2 and baseline low-density lipoprotein cholesterol levels;

^||^Model 4: adjusted for all factors in model 3 and usage of antiplatelet, antihypertensive, hypoglycemic and statin during 1-year follow-up period

After classifying patients according to the TOAST criteria, the highest quartiles of IL-6 and YKL-40 were associated with increased risk of recurrent stroke only in the patients with large-artery atherosclerosis (IL-6: HR, 1.66; 95% CI 1.21–2.29; *P* = 0.002; YKL-40: HR, 1.81; 95% CI 1.32–2.48; *P* = 0.0002) and small-vessel occlusion (IL-6: HR 1.67; 95% CI 1.06–2.64; *P* = 0.03; YKL-40: HR 1.84; 95% CI 1.17–2.88; *P* = 0.008) subtypes (Additional file 1: Table S11). In the multivariate model, such correlation of YKL-40 with recurrent stroke persisted in these two stroke subtypes (adjusted HR 1.46; 95% CI 1.03–2.06; *P* = 0.03 and HR, 1.89; 95% CI 1.12–3.18; *P* = 0.02, respectively), while no statistical significance received for IL-6 (Additional file 1: Table S11).

### Markers and poor functional outcome

A total of 2395 (23.4%) patients had poor functional outcome within 1 year. The highest quartiles of IL-6 (adjusted OR 1.93; 95% CI 1.64–2.27; *P* < 0.0001), IL-1Ra (adjusted OR 1.60; 95% CI 1.37–1.87; *P* < 0.0001), hsCRP (adjusted OR 1.60; 95% CI 1.37–1.86; *P* < 0.0001) and YKL-40 (adjusted OR 1.21; 95% CI 1.03–1.42; *P* = 0.02) were associated with increased risk of poor functional outcome (Table 3). Similar results were found for the shifts in the distributions of the mRS scores (Additional file 1: Fig. S5). In the sensitivity analysis, results were consistent for the outcome of dependence or death (mRS ≥ 3) (Additional file 1: Table S12). In the multivariate stepwise regression analysis, among the inflammatory markers, the associations between IL-6 (OR 1.68; 95% CI 1.46–1.93; *P* < 0.0001) or YKL-40 (OR 1.02; 95% CI 1.01–1.03; *P* = 0.0001) and poor functional outcome were maintained (Additional file 1: Fig. S6). ROC analysis indicated that the optimal cut-off values for IL-6 and YKL-40 were 3.22 ng/L and 75.82 mg/L, respectively.

**Table 3:** Associations of individual inflammation marker or combination of IL-6 and YKL-40 with a Modified Rankin Scale score ≥ 2 within 1 year

Marker and levels *		Events [no., (%)]	Model 1^†^		Model 2^‡^		Model 3^§^		Model 4^||^	
			OR (95% CI)	*P* value	OR (95% CI)	*P* value	OR (95% CI)	*P* value	OR (95% CI)	*P* value
IL-6 (ng/L)	Q1	336 (13.0)	Reference	–	Reference	–	Reference	–	Reference	–
	Q2	500 (19.5)	1.62 (1.39–1.88)	< 0.0001	1.32 (1.13–1.55)	0.0006	1.34 (1.15–1.58)	0.0003	1.39 (1.17–1.63)	0.0001
	Q3	626 (24.5)	2.16 (1.87–2.50)	< 0.0001	1.44 (1.23–1.68)	< 0.0001	1.47 (1.26–1.72)	< 0.0001	1.48 (1.26–1.74)	< 0.0001
	Q4	933 (37.0)	3.92 (3.41–4.51)	< 0.0001	1.88 (1.61–2.20)	< 0.0001	1.95 (1.66–2.28)	< 0.0001	1.93 (1.64–2.27)	< 0.0001
	Continuous model		1.82 (1.73–1.92)	< 0.0001	1.32 (1.24–1.41)	< 0.0001	1.34 (1.26–1.43)	< 0.0001	1.32 (1.24–1.41)	< 0.0001
IL-1Ra (ng/L)	Q1	462 (18.0)	Reference	–	Reference	–	Reference	–	Reference	–
	Q2	504 (19.7)	1.11 (0.97–1.28)	0.14	1.00 (0.87–1.17)	0.97	1.01 (0.86–1.17)	0.93	1.00 (0.86–1.17)	0.98
	Q3	638 (24.9)	1.51 (1.32–1.72)	< 0.0001	1.24 (1.07–1.44)	0.005	1.24 (1.07–1.45)	0.004	1.25 (1.07–1.46)	0.006
	Q4	791 (31.3)	2.07 (1.81–2.36)	< 0.0001	1.55 (1.34–1.80)	< 0.0001	1.54 (1.32–1.78)	< 0.0001	1.60 (1.37–1.87)	< 0.0001
	Continuous model		1.51 (1.41–1.63)	< 0.0001	1.32 (1.21–1.43)	< 0.0001	1.31 (1.20–1.42)	< 0.0001	1.33 (1.22–1.45)	< 0.0001
hsCRP (mg/L)	Q1	422 (16.3)	Reference	–	Reference	–	Reference	–	Reference	–
	Q2	460 (18.1)	1.14 (0.98–1.31)	0.08	1.06 (0.91–1.24)	0.43	1.06 (0.91–1.24)	0.43	1.07 (0.91–1.25)	0.43
	Q3	606 (23.8)	1.60 (1.40–1.84)	< 0.0001	1.30 (1.12–1.51)	0.0005	1.32 (1.14–1.53)	0.0003	1.29 (1.11–1.51)	0.001
	Q4	907 (35.9)	2.88 (2.52–3.28)	< 0.0001	1.62 (1.40–1.87)	< 0.0001	1.65 (1.42–1.91)	< 0.0001	1.60 (1.37–1.86)	< 0.0001
	Continuous model		1.38 (1.33–1.42)	< 0.0001	1.16 (1.11–1.20)	< 0.0001	1.16 (1.12–1.21)	< 0.0001	1.15 (1.11–1.20)	< 0.0001
Lp-PLA_2_ (μg/L)	Q1	582 (22.8)	Reference	–	Reference	–	Reference	–	Reference	–
	Q2	564 (22.2)	0.97 (0.85–1.10)	0.61	0.98 (0.84–1.13)	0.74	0.98 (0.84–1.13)	0.73	0.97 (0.84–1.13)	0.73
	Q3	609 (23.7)	1.06 (0.93–1.20)	0.41	1.07 (0.93–1.23)	0.36	1.06 (0.92–1.23)	0.41	1.04 (0.90–1.21)	0.61
	Q4	640 (25.1)	1.14 (1.00–1.30)	0.05	1.08 (0.94–1.25)	0.26	1.08 (0.94–1.25)	0.29	1.05 (0.91–1.22)	0.50
	Continuous model		1.11 (1.00–1.23)	0.06	1.07 (0.96–1.20)	0.24	1.07 (0.95–1.20)	0.25	1.04 (0.93–1.18)	0.49
Lp-PLA_2_-A (nmol/min/ml)	Q1	586 (22.9)	Reference	–	Reference	–	Reference	–	Reference	–
	Q2	567 (22.1)	0.96 (0.84–1.09)	0.50	0.97 (0.84–1.12)	0.69	0.98 (0.85–1.13)	0.75	0.97 (0.84–1.13)	0.71
	Q3	588 (23.0)	1.00 (0.88–1.14)	0.95	0.97 (0.84–1.12)	0.64	0.97 (0.84–1.12)	0.65	0.93 (0.80–1.08)	0.32
	Q4	654 (25.8)	1.17 (1.03–1.33)	0.02	1.13 (0.98–1.30)	0.10	1.13 (0.98–1.30)	0.10	1.09 (0.94–1.26)	0.25
	Continuous model		1.14 (0.99–1.30)	0.06	1.09 (0.94–1.27)	0.23	1.10 (0.94–1.27)	0.23	1.05 (0.90–1.22)	0.55
YKL-40 (mg/L)	Q1	445 (17.5)	Reference	–	Reference	–	Reference	–	Reference	–
	Q2	537 (20.9)	1.25 (1.09–1.44)	0.002	1.06 (0.91–1.23)	0.47	1.07 (0.92–1.24)	0.41	1.07 (0.91–1.25)	0.44
	Q3	585 (22.9)	1.40 (1.22–1.61)	< 0.0001	0.95 (0.81–1.11)	0.48	0.96 (0.82–1.12)	0.58	0.94 (0.80–1.11)	0.48
	Q4	828 (32.6)	2.29 (2.00–2.61)	< 0.0001	1.20 (1.03–1.40)	0.02	1.24 (1.06–1.45)	0.007	1.21 (1.03–1.42)	0.02
	Continuous model		1.54 (1.45–1.64)	< 0.0001	1.10 (1.02–1.19)	0.01	1.12 (1.04–1.21)	0.003	1.11 (1.02–1.20)	0.01
IL-6 + YKL-40	Q1	477 (15.2)	Reference	–	Reference	–	Reference	–	Reference	–
	Q2	359 (18.0)	1.23 (1.05–1.42)	0.008	0.91 (0.78–1.08)	0.28	0.93 (0.79–1.09)	0.34	0.92 (0.78–1.09)	0.33
	Q3	505 (25.6)	1.92 (1.67–2.21)	< 0.0001	1.31 (1.13–1.53)	0.0005	1.33 (1.14–1.55)	0.0002	1.33 (1.13–1.55)	0.0005
	Q4	1054 (34.0)	2.88 (2.54–3.25)	< 0.0001	1.36 (1.18–1.57)	< 0.0001	1.40 (1.22–1.62)	< 0.0001	1.36 (1.17–1.58)	< 0.0001

*OR* odds ratio, *CI* confidence intervals, *Q1* quartile 1, *Q2* quartile 2, *Q3* quartile 3, *Q4* quartile 4, *IL-6* interleukin-6, *IL-1Ra* interleukin-1 receptor antagonist, *hsCRP* high sensitive C-reactive protein, *Lp-PLA*_*2*_ lipoprotein-associated phospholipase A_2_, *Lp-PLA*_*2*_*-A* lipoprotein-associated phospholipase A_2_ activity

*All markers were categorized into 4 even groups by quartiles. In the continuous model, the odds ratios correspond to per-unit increment of logarithm of marker value

^†^Model 1: unadjusted

^‡^Model 2: adjusted for age, sex, body mass index, smoking, medical histories of atrial fibrillation, coronary heart disease, ischemic stroke, diabetes, hypertension and hypercholesterolemia, baseline NIHSS score, mRS score before the onset of index events and baseline leukocyte count

^§^Model 3: adjusted for all factors in Model 2 and tPA treatment

^||^Model 4: adjusted for all factors in Model 3 and stroke recurrence within 1 year

### Predictive value of IL-6 in combination with YKL-40

Based on the results of multivariate stepwise regression analyses, we further assessed the potential gains in predictive value by combining IL-6 and YKL-40 levels. Patients with elevated levels of both IL-6 and YKL-40 also had increased risk of recurrent stroke (adjusted HR, 1.37; 95% CI 1.14–1.63; *P* = 0.0007), poor functional outcome (adjusted OR 1.36; 95% CI 1.17–1.58; *P* < 0.0001) and dependence or death (adjusted OR 1.91; 95% CI 1.56–2.34; *P* < 0.0001) (Tables 2, 3 and Additional file 1: Table S12).

Adding IL-6 and YKL-40 significantly increased the area under the ROC curves for the prediction models of Essen Stroke Risk Score (0.03, *P* < 0.0001) and Totaled Health Risks in Vascular Events Score (0.07, *P* < 0.0001), as well as resulted in continuous net reclassification index (19.0%, *P* < 0.0001; 33.0, *P* < 0.0001) (Table 4).

**Table 4:** Reclassification and discrimination statistics for clinical outcomes by adding inflammatory markers of IL-6 and YKL-40 to clinical risk scores

Outcomes	Model	AUC		NRI,%	
		Estimate (95% CI)	*P* value	Estimate (95% CI)	*P* value
Stroke recurrence	ESRS	0.54 (0.52–0.55)	–	Reference	–
	ESSEN + IL-6 + YKL-40	0.57 (0.55–0.58)	< 0.0001	19.0 (12.7–25.4)	< 0.0001
mRS (2–6)	THRIVE score	0.57 (0.56–0.58)	–	Reference	–
	THRIVE Score + IL-6 + YKL-40	0.64 (0.63–0.66)	< 0.0001	33.0 (28.4–37.6)	< 0.0001

*AUC* area under the curve, *CI* confidence interval, *NRI* net reclassification index, *ESRS* Essen Stroke Risk Score, *THRIVE Score* totaled health risks in vascular events score

## Discussion

Instead of focusing on a single inflammatory molecule, we analyzed multiple atherosclerotic inflammatory biomarkers in the current large-scaled multicenter and prospective study, thus providing a comprehensive picture of systemic and vascular inflammation after stroke. We found that the predictive value of IL-6 and YKL-40 in recurrent stroke, composite vascular event and poor functional outcome among the patients with acute ischemic stroke or TIA was more apparent than that of hsCRP and Lp-PLA2 mass and activity.

Different mechanisms might exist for these associations. As an acute-phase reactant produced by hepatocytes, IL-6 has a variety of functions, including propagating the downstream inflammatory response through initiating the Janus kinase and signal transducer and activator of transcription signaling pathways, which contributes to cerebral damage and exerts compact on neurological function [24], and activation of endothelial cells, increased coagulation, and promotion of lymphocyte proliferation and differentiation, and IL-6 has been implicated in progression of atherosclerosis and plaque instability, which may explain how it impacts the development of subsequent stroke [25, 26]. From the point of view of vascular biology, YKL-40 has been shown to activate both mitogen-activated protein kinase and phosphatidylinositol 3′-kinase, which modulates cell proliferation, survival, migration and adhesion, and reflect local vascular bed atherosclerotic inflammation, which consequently might affect the occurrence of vascular event [27–29]. Among the five stroke subtypes classified according to TOAST criteria, large-artery atherosclerosis and small-vessel occlusion have several risk factors in common that largely contribute to atherosclerosis. Focal inflammation and atherosclerosis have been suggested to be the main pathophysiology of these two subtypes [30]. In our study, we found that the correlations of IL-6 and YKL-40 with recurrent stroke were more apparent in the patients with large-artery atherosclerosis and small-vessel occlusion, implying the association between IL-6 and YKL-40 and atherosclerosis progression, which possibly lead to stroke recurrence. However, the correlations disappeared for IL-6 after adjusting for confounders. Since sample size reduced after TOAST classification, insufficient sample size might be one cause. Further study was needed to verify our results.

On the other hand, though prior Mendelian randomization studies had found that YKL-40 did not play a causative role in the development of cardiovascular disease, [31, 32] these results did not exclude the possibility that YKL-40 could play a role in disease progression, even recurrence. Some previous population-based studies showed that YKL-40 was associated with first stroke, but not myocardial infarction [8, 33]. Moreover, in a European population with stable coronary artery disease, YKL-40 was shown to be associated with composite vascular events, but not cerebrovascular disease after adjusting for other risk factors [34, 35]. These results might suggest the pathophysiological importance of YKL-40 in susceptible vascular bed, and its role as a biomarker reflecting plaque development primarily elicited by risk factor presence in itself, which was consistent with our findings that comorbidity and presence of risk factors were more common in the patients with elevated levels of YKL-40 and YKL-40 predicted recurrence more apparently in the patients with large-artery atherosclerosis. Our results added evidence regarding the positive role of YKL-40 in recurrence after ischemic stroke. On the other hand, it has been shown that YKL-40 could be induced by the cytokine of IL-1β [36], which might explain the positive though relatively weak correlation between YKL-40 and IL-6 in our study. We therefore applied multivariate stepwise regression analyses to reveal the relative usefulness of each marker. When simultaneously considering all markers, IL-6 and YKL-40 remained predictive for recurrent stroke and poor functional outcome. The addition of IL-6 and YKL-40 modestly increased the ROCs of ESSEN and THRIVE models and led to a highly significant NRIs. However, comparing with IL-6 alone, the improvement of predictive value of combining IL-6 and YKL-40 was mild, suggesting the domain role of IL-6.

It has been noted that the prognosis of recurrent stroke is unfavorable, and in China, stroke has ranked third among the leading causes of death behind malignant tumors and heart disease [37, 38]. We therefore adjusted for stroke recurrence to exclude the possibility that poor functional outcome was resulted in by recurrence. The predictive accuracy of Essen Stroke Risk Score and the Totaled Health Risks in Vascular Events Score was lower than that in the previous study [22, 23], and the improvement by adding inflammatory markers was significantly moderate, which might be mainly due to improvement of secondary prevention management of ischemic cerebrovascular disease, proved by 38% decrease (9.9% vs. 16%) in the rate of stroke recurrence in the current study [22]. We believe our data at least have potential importance for risk stratification of patients as well as the design of future trials when choosing systemic or local inflammation markers or treatment targets.

On the other hand, the residual risk of recurrent stroke and death is substantial despite of early management and administrating secondary prevention therapy, calling for measures from other perspective. Atherosclerotic plaque inflammation has been suggested to be an important contributor to plaque destabilization and thromboembolic events. In our study, the relationship between inflammatory markers and recurrent stroke persisted even after adjusting for currently available secondary preventions, including statin and antiplatelet, antihypertensive and hypoglycemic agents. The CANTOS and LoDoCo2 (Low-Dose Colchicine for Secondary Prevention of Cardiovascular Disease 2) studies have demonstrated the protective effect of anti-inflammatory treatment in the patients with coronary heart diseases [10, 39]. However, little was known for stroke prevention. The ongoing CONVINCE (Colchicine for Prevention of Vascular Inflammation in Non-cardio Embolic Stroke) study evaluating the effect of low-dose of colchicine in reducing the rate of recurrent stroke in patients with stroke would shed some light on it [40]. Moreover, our findings also have implications for patients care. Beyond pharmacologic interventions, lifestyle such as diet, exercise and smoking cessation all lowered vascular inflammation. Therefore, lifestyle modification should be emphasized as well, and measurements of inflammation marker levels could be used to motivate lifestyle choices.

IL-1β induces the production of IL-1Ra, which is an endogenous inhibitor of IL-1β [41]. Since a direct measurement of IL-1β was not applicable due to extremely low circulation levels, IL-1Ra could serve as a detectable surrogate parameter for high IL-1β activity [42]. Our data regarding the positive association between IL-1Ra and poor functional outcome merited careful consideration. As an antagonist of IL-1β, IL-1Ra treatment has been highly neuroprotective experimentally [43], while endogenous levels of IL-1Ra have been elucidated to be upregulated and related to adverse outcome after cardiovascular disease and stroke [4, 5]. The functional role of IL-1Ra in the context of atherogenesis has yet to be fully determined [44]. In this regard, two major assumptions might be postulated. First, elevated IL-Ra levels might be atheroprotective, as an insufficient attempt by the body to counter-regulate the concomitant increases of IL-1β activity [45]. Therefore, IL-1Ra might mainly serve as an indirect indicator for IL-1β. Second, IL-1Ra itself might induce atherosclerosis. A large genetic study has found that IL-1Ra-rising alleles lead to an increased cardiovascular risk [46]. However, the hypothesis that binding of IL-1Ra to the IL-1 receptor triggered harmful downstream signaling still needed to be verified. Moreover, given the result of the CANTOS trial, which lowered rate of recurrent cardiovascular event by using anti-inflammatory therapy targeting interleukin-1β, [10] the latter assumption became less likely, but, on the other hand, cannot be totally excluded.

Our study also had several limitations. First, only one point measurements of markers were available, incapacitating us to evaluate the effect of changes in the levels of these markers over time. Second, we did not collect information about how the traditional risk factors were controlled during follow-up under the secondary prevention treatment. Third, though centralized etiology classification of index event was performed, acquirement of stroke subtypes of recurrent stroke would be informative. Fourth, all participants were Chinese, thus, our findings may not be generalizable to other races and ethnicities.

## Conclusions

In the patients with ischemic stroke or TIA, we found independent associations of IL-6 or YKL-40 with recurrent stroke, composite vascular events and poor functional outcome, which were more apparent than that of hsCRP and Lp-PLA2 mass and activity. The addition of IL-6 and YKL-40 improved risk classification of clinical risk algorithms. Our findings provided a comprehensive picture of systemic and vascular inflammation after stroke, helped optimizing risk stratification and might shed some light on choosing inflammation target when designing the clinical trials of stroke in the future.

## Supplementary Information

**Additional file 1.** Supplemental tables and figures on participant selection, baseline characteristics and the associations of markers with outcomes.

## Data Availability

All data and materials are available to researchers on request for purposes of reproducing the results or replicating the procedure by directly contacting the corresponding author.

## Abbreviations

IL-6: Interleukin-6
IL-1Ra: Interleukin-1 receptor antagonist
hsCRP: High sensitive C-reactive protein
Lp-PLA_2_: Lipoprotein-associated phospholipase A_2_
Lp-PLA_2_-A: Lipoprotein-associated phospholipase A_2_ activity
CNSR-III: The Third China National Stroke Registry
TIA: Transient ischemic attack
mRS: Modified Rankin Scale
CANTOS: The Canakinumab Antiinflammatory Thrombosis Outcome Study
COLCOT: The Colchicine Cardiovascular Outcomes Trial
mRS: Modified Rankin Scale
NIHSS: National Institutes of Health Stroke Scale
BMI: Body mass index
HR: Hazard ratio
OR: Odds ratio
CI: Confidence intervals
Q1: Quartile 1
Q2: Quartile 2
Q3: Quartile 3
Q4: Quartile 4
