# Supplementary material for: Interleukin-6 and YKL-40 predicted recurrent stroke after ischemic stroke or TIA: analysis of 6 inflammation biomarkers in a prospective cohort study
Source: J Neuroinflammation. 2022 Jun 6;19:131. doi: 10.1186/s12974-022-02467-1 (PMC9235241; doi:10.1186/s12974-022-02467-1)

Additional Table 1. Characteristics of Patients Included versus not Included in Inflammatory Biomarker Substudy of the CNSR-III.

|  | Patients included  n=10,472 | Not included  n=4694 | P value |
| --- | --- | --- | --- |
| Age (year), median (IQR) | 63 (54-70) | 62 (54-70) | 0.33 |
| Male, No. (%) | 7165 (68.4) | 3199 (68.2) | 0.74 |
| Body mass index, median (IQR) | 24.5 (22.6-26.6) | 24.5 (22.7-26.5) | 0.65 |
| Smoking, No. (%) | 4654 (44.4) | 2006 (42.7) | 0.06 |
| Medical history, No. (%) |  |  |  |
| Ischemic stroke | 2237 (21.4) | 912 (19.4) | 0.007 |
| Diabetes | 2510 (24.0) | 1000 (21.3) | 0.0003 |
| Hypertension | 6586 (62.9) | 2908 (62.0) | 0.28 |
| Hypercholesterolemia | 869 (8.3) | 322 (6.9) | 0.002 |
| Coronary heart disease | 1135 (10.8) | 473 (10.1) | 0.16 |
| Atrial fibrillation | 753 (7.2) | 266 (5.7) | 0.0005 |
| Index event, No. (%) |  |  |  |
| Ischemic stroke | 9753 (93.1) | 4393 (93.6) | 0.35 |
| TIA | 719 (6.9) | 301 (6.4) | 0.30 |
| mRS score before the onset of index events≥2, No. (%) | 920 (8.8) | 424 (9.0) | 0.62 |
| Leukocyte count (*10^9^/L), median (IQR) | 6.9 (5.7-8.4) | 6.9 (5.8-8.4) | 0.55 |

Abbreviation: CNSR-III =The Third China National Stroke Registry; IQR = interquartile range; TIA= transient ischemic attack; mRS=modified Rankin Scale.

Additional Table 2. Distribution of Baseline Characteristics According to IL-6 Quartiles

|  | IL-6 levels (ng/L) | | | | P value |
| --- | --- | --- | --- | --- | --- |
|  | ≤1.6 | 1.6 to 2.6 | 2.6 to 5.1 | >5.1 |  |
| Age (year), median (IQR) | 59 (52-66) | 62 (54-68) | 64 (56-71) | 67 (59-75) | <0.0001 |
| Male, No. (%) | 1853 (70.7) | 1770 (67.7) | 1759 (67.2) | 1783 (68.1) | 0.03 |
| Body mass index, median (IQR) | 24.4 (22.5-26.3) | 24.7 (22.9-26.8) | 24.5 (22.6-26.6) | 24.2 (22.3-26.4) | 0.0003 |
| Smoking, No. (%) | 1159 (44.2) | 1154 (44.1) | 1211 (46.3) | 1130 (43.2) | 0.15 |
| Medical history, No. (%) |  |  |  |  |  |
| Ischemic stroke | 429 (16.4) | 556 (21.3) | 609 (23.3) | 643 (24.6) | <0.0001 |
| Diabetes | 530 (20.2) | 655 (25.0) | 707 (27.0) | 618 (23.6) | <0.0001 |
| Hypertension | 1532 (58.5) | 1679 (64.2) | 1691 (64.6) | 1684 (64.3) | <0.0001 |
| Hypercholesterolemia | 197 (7.5) | 222 (8.5) | 250 (9.6) | 200 (7.6) | 0.03 |
| Coronary heart disease | 181 (6.9) | 266 (10.2) | 315 (12.0) | 373 (14.3) | <0.0001 |
| Atrial fibrillation | 70 (2.7) | 116 (4.4) | 192 (7.3) | 375 (14.3) | <0.0001 |
| Index event, No. (%) |  |  |  |  |  |
| Ischemic stroke | 2339 (89.3) | 2429 (92.9) | 2476 (94.6) | 2509 (95.8) | <0.0001 |
| TIA | 281 (10.7) | 187 (7.2) | 142 (5.4) | 109 (4.2) | <0.0001 |
| Baseline NIHSS, median (IQR) | 2 (1-4) | 3 (1-5) | 3 (2-6) | 5 (2-8) | <0.0001 |
| mRS score before the onset of index events≥2, No. (%) | 164 (6.3) | 209 (8.0) | 233 (8.9) | 314 (12.0) | <0.0001 |
| Leukocyte count (*10^9^/L), median (IQR) | 6.5 (5.4-7.8) | 6.8 (5.7-8.1) | 7.0 (5.8-8.4) | 7.5 (6.1-9.2) | 0.02 |
| LDL-C levels (mmol/L), median (IQR) | 2.4 (1.8-3.0) | 2.3 (1.7-3.0) | 2.3 (1.7-3.0) | 2.3 (1.7-2.9) | 0.005 |

Abbreviation: IL-6=interleukin-6; IQR = interquartile range; NIHSS=National Institutes of Health Stroke Scale; mRS=modified Rankin Scale; LDL-C, low-density lipoprotein cholesterol.

Additional Table 3. Distribution of Baseline Characteristics According to IL-1Ra Quartiles

|  | IL-1Ra levels (ng/L) | | | | P value |
| --- | --- | --- | --- | --- | --- |
|  | ≤254.6 | 254.6 to 341.3 | 341.3 to 497.8 | >497.8 |  |
| Age, median (IQR) | 61 (54-69) | 62 (54-70) | 63 (54-71) | 64 (55-72) | <0.0001 |
| Male, No. (%) | 2095 (80.0) | 1891 (72.2) | 1703 (65.1) | 1476 (56.4) | <0.0001 |
| Body mass index, median (IQR) | 23.9 (22.0-25.8) | 24.4 (22.5-26.4) | 24.8 (22.9-26.9) | 25.0 (23.0-27.3) | <0.0001 |
| Smoking, No. (%) | 1368 (52.3) | 1211 (46.3) | 1133 (43.3) | 942 (36.0) | <0.0001 |
| Medical history, No. (%) |  |  |  |  |  |
| Ischemic stroke | 535 (20.4) | 547 (20.9) | 588 (22.5) | 567 (21.7) | 0.30 |
| Diabetes | 495 (18.9) | 624 (23.8) | 667 (25.5) | 724 (27.7) | <0.0001 |
| Hypertension | 1482 (56.6) | 1662 (63.5) | 1669 (63.8) | 1773 (67.7) | <0.0001 |
| Hypercholesterolemia | 180 (6.9) | 219 (8.4) | 235 (9.0) | 235 (9.0) | 0.02 |
| Coronary heart disease | 220 (8.4) | 279 (10.7) | 304 (11.6) | 332 (12.7) | <0.0001 |
| Atrial fibrillation | 148 (5.7) | 166 (6.3) | 201 (7.7) | 238 (9.1) | <0.0001 |
| Index event, No. (%) |  |  |  |  |  |
| Ischemic stroke | 2382 (91.0) | 2430 (92.8) | 2472 (94.4) | 2469 (94.3) | <0.0001 |
| TIA | 236 (9.0) | 188 (7.2) | 146 (5.6) | 149 (5.7) | <0.0001 |
| Baseline NIHSS, median (IQR) | 3 (1-5) | 3 (1-6) | 3 (1-6) | 4 (2-7) | <0.0001 |
| mRS score before the onset of index events≥2, No. (%) | 185 (7.1) | 214 (8.2) | 232 (8.7) | 289 (11.0) | <0.0001 |
| Leukocyte count (*10^9^/L), median (IQR) | 6.4 (5.3-7.7) | 6.8 (5.7-8.2) | 7.1 (5.9-8.6) | 7.4 (6.1-9.0) | <0.0001 |
| LDL-C levels (mmol/L), median (IQR) | 2.4 (1.8-3.1) | 2.4 (1.7-3.0) | 2.3 (1.7-3.0) | 2.3 (1.7-3.0) | 0.0002 |

Abbreviation: IL-1Ra = interleukin-1 receptor antagonist; IQR = interquartile range; NIHSS=National Institutes of Health Stroke Scale; mRS=modified Rankin Scale; LDL-C, low-density lipoprotein cholesterol.

Additional Table 4. Distribution of Baseline Characteristics According to hsCRP Levels

|  | hsCRP levels (mg/L) | | | | P value |
| --- | --- | --- | --- | --- | --- |
|  | ≤0.8 | 0.8 to 1.8 | 1.8 to 4.7 | >4.7 |  |
| Age, median (IQR) | 61 (53-68) | 61 (54-68) | 62 (54-70) | 65 (58-74) | <0.0001 |
| Male, No. (%) | 1883 (71.5) | 1786 (68.6) | 1727 (66.0) | 1769 (67.6) | 0.0002 |
| Body mass index, median (IQR) | 24.2 (22.2-26.0) | 24.6 (22.8-26.6) | 24.8 (22.9-27.1) | 24.5 (22.5-26.6) | <0.0001 |
| Smoking, No. (%) | 1215 (46.1) | 1158 (44.5) | 1146 (43.8) | 1135 (43.4) | 0.20 |
| Medical history, No. (%) |  |  |  |  |  |
| Ischemic stroke | 534 (20.3) | 527 (20.3) | 538 (20.6) | 638 (24.4) | 0.0003 |
| Diabetes | 577 (21.9) | 579 (22.2) | 705 (26.9) | 649 (24.8) | <0.0001 |
| Hypertension | 1557 (59.1) | 1643 (63.1) | 1682 (64.3) | 1704 (65.1) | <0.0001 |
| Hypercholesterolemia | 204 (7.7) | 210 (8.1) | 252 (9.6) | 203 (7.8) | 0.04 |
| Coronary heart disease | 222 (8.4) | 233 (9.0) | 321 (12.3) | 359 (13.7) | <0.0001 |
| Atrial fibrillation | 99 (3.8) | 144 (5.5) | 177 (6.8) | 333 (12.7) | <0.0001 |
| Index event, No. (%) |  |  |  |  |  |
| Ischemic | 2391 (90.7) | 2402 (92.3) | 2463 (94.1) | 2497 (95.4) | <0.0001 |
| TIA | 244 (9.3) | 201 (7.7) | 154 (5.9) | 120 (4.6) | <0.0001 |
| Baseline NIHSS, median (IQR) | 3 (1-5) | 3 (1-5) | 3 (1-6) | 4 (2-8) | <0.0001 |
| mRS score before the onset of index events≥2, No. (%) | 200 (7.6) | 194 (7.5) | 224 (8.6) | 302 (11.5) | <0.0001 |
| Leukocyte count (*10^9^/L), median (IQR) | 6.3 (5.3-7.5) | 6.7 (5.6-8.1) | 7.1 (5.9-8.6) | 7.7 (6.3-9.4) | <0.0001 |
| LDL-C levels (mmol/L), median (IQR) | 2.2 (1.7-2.9) | 2.4 (1.7-3.0) | 2.4 (1.8-3.1) | 2.4 (1.8-3.0) | <0.0001 |

Abbreviation: hsCRP= high sensitive C-reactive protein; IQR = interquartile range; NIHSS=National Institutes of Health Stroke Scale; mRS=modified Rankin Scale; LDL-C, low-density lipoprotein cholesterol.

Additional Table 5. Distribution of Baseline Characteristics According to Lp-PLA_2_ Quartiles

|  | Lp-PLA_2_ levels (ug/L) | | | | P value |
| --- | --- | --- | --- | --- | --- |
|  | ≤127.6 | 127.6 to 175.0 | 175.0 to 225.1 | >225.1 |  |
| Age, median (IQR) | 62 (54-70) | 63 (54-70) | 62 (54-70) | 63 (55-71) | 0.002 |
| Male, No. (%) | 1809 (69.1) | 1763 (67.4) | 1797 (68.6) | 1796 (68.6) | 0.60 |
| Body mass index, median (IQR) | 24.2 (22.5-26.2) | 24.6 (22.6-26.7) | 24.5 (22.7-26.6) | 24.5 (22.6-26.6) | 0.02 |
| Smoking, No. (%) | 1125 (42.9) | 1143 (43.7) | 1173 (44.8) | 1213 (46.3) | 0.07 |
| Medical history, No. (%) |  |  |  |  |  |
| Ischemic stroke | 609 (23.2) | 572 (21.9) | 531 (20.3) | 525 (20.0) | 0.02 |
| Diabetes | 672 (25.7) | 574 (21.9) | 622 (23.8) | 642 (24.5) | 0.02 |
| Hypertension | 1657 (63.2) | 1644 (62.8) | 1637 (62.5) | 1648 (63.0) | 0.96 |
| Hypercholesterolemia | 191 (7.3) | 225 (8.6) | 227 (8.7) | 226 (8.6) | 0.20 |
| Coronary heart disease | 264 (10.1) | 287 (11.0) | 299 (11.4) | 285 (10.9) | 0.47 |
| Atrial fibrillation | 181 (6.9) | 183 (7.0) | 186 (7.1) | 203 (7.8) | 0.63 |
| Index event, No. (%) |  |  |  |  |  |
| Ischemic stroke | 2450 (93.5) | 2406 (92.0) | 2428 (92.7) | 2469 (94.3) | 0.006 |
| TIA | 174 (6.3) | 233 (8.4) | 201 (7.2) | 160 (6.8) | 0.0006 |
| Baseline NIHSS, median (IQR) | 3 (1-6) | 3 (1-6) | 3 (1-6) | 3 (1-6) | 0.006 |
| mRS score before the onset of index events≥2, No. (%) | 258 (9.9) | 225 (8.6) | 209 (8.0) | 228 (8.7) | 0.12 |
| Leukocyte count (*10^9^/L), median (IQR) | 6.9 (5.6-8.4) | 6.9 (5.7-8.4) | 6.9 (5.7-8.4) | 7.0 (5.8-8.5) | 0.24 |
| LDL-C levels (mmol/L), median (IQR) | 1.9 (1.4-2.4) | 2.1 (1.6-2.7) | 2.4 (1.9-3.0) | 2.9 (2.4-3.6) | <0.0001 |

Abbreviation: Lp-PLA2= lipoprotein-associated phospolipase A2; IQR = interquartile range; NIHSS=National Institutes of Health Stroke Scale; mRS=modified Rankin Scale; LDL-C, low-density lipoprotein cholesterol.

Additional Table 6. Distribution of Baseline Characteristics According to Lp-PLA_2_-A Quartiles

|  | Lp-PLA_2_-A levels (nmol/min/ml) | | | | P value |
| --- | --- | --- | --- | --- | --- |
|  | ≤128.0 | 128.0 to 162.2 | 162.2 to 194.6 | >194.6 |  |
| Age, median (IQR) | 63 (55-70) | 62 (54-71) | 62 (54-70) | 63 (55-71) | 0.38 |
| Male, No. (%) | 1645 (62.8) | 1659 (63.3) | 1832 (70.0) | 2029 (77.6) | <0.0001 |
| Body mass index, median (IQR) | 24.3 (22.5-26.4) | 24.3 (22.5-26.3) | 24.7 (22.7-26.8) | 24.5 (22.8-26.6) | 0.001 |
| Smoking, No. (%) | 1051 (40.2) | 1086 (41.4) | 1149 (43.9) | 1368 (52.3) | <0.0001 |
| Medical history, No. (%) |  |  |  |  |  |
| Ischemic stroke | 631 (24.1) | 562 (21.4) | 520 (19.9) | 524 (20.1) | 0.0005 |
| Diabetes | 666 (25.4) | 654 (25.0) | 606 (23.1) | 584 (22.3) | 0.03 |
| Hypertension | 1672 (63.9) | 1644 (62.7) | 1661 (63.4) | 1609 (61.6) | 0.33 |
| Hypercholesterolemia | 207 (7.9) | 230 (8.8) | 220 (8.4) | 212 (8.1) | 0.69 |
| Coronary heart disease | 255 (9.7) | 316 (12.1) | 260 (9.9) | 304 (11.6) | 0.01 |
| Atrial fibrillation | 177 (6.8) | 186 (7.1) | 199 (7.6) | 191 (7.3) | 0.69 |
| Index event, No. (%) |  |  |  |  |  |
| Ischemic stroke | 2419 (92.4) | 2416 (92.2) | 2459 (93.9) | 2459 (94.1) | 0.008 |
| TIA | 199 (7.6) | 205 (7.8) | 160 (6.1) | 155 (5.9) | 0.008 |
| Baseline NIHSS, median (IQR) | 3 (1-6) | 3 (1-5) | 3 (1-6) | 3 (2-6) | <0.0001 |
| mRS score before the onset of index events≥2, No. (%) | 253 (9.7) | 193 (7.4) | 241 (9.2) | 233 (8.9) | 0.02 |
| Leukocyte count (*10^9^/L), median (IQR) | 6.8 (5.6-8.3) | 6.9 (5.7-8.4) | 6.9 (5.7-8.4) | 7.1 (5.9-8.6) | 0.0001 |
| LDL-C levels (mmol/L), median (IQR) | 1.8 (1.3-2.4) | 2.1 (1.6-2.6) | 2.5 (2.0-3.1) | 2.9 (2.3-3.5) | <0.0001 |

Abbreviation: Lp-PLA_2_-A= lipoprotein-associated phospolipase A_2_ activity; IQR = interquartile range; NIHSS=National Institutes of Health Stroke Scale; mRS=modified Rankin Scale; LDL-C, low-density lipoprotein cholesterol.

Additional Table 7. Distribution of Baseline Characteristics According to YKL-40 Quartiles

|  | YKL-40 levels (mg/L) | | | | P value |
| --- | --- | --- | --- | --- | --- |
|  | ≤38.1 | 38.1 to 65.3 | 65.3 to 123.3 | >123.3 |  |
| Age, median (IQR) | 56 (49-63) | 61 (53-67) | 65 (58-72) | 69 (62-76) | <0.0001 |
| Male, No. (%) | 2003 (76.5) | 1863 (71.2) | 1766 (67.5) | 1533 (58.6) | <0.0001 |
| Body mass index, median (IQR) | 24.8 (23.0-26.8) | 24.7 (22.9-26.7) | 24.4 (22.5-26.4) | 24.2 (22.0-26.1) | <0.0001 |
| Smoking, No. (%) | 1350 (48.4) | 1255 (47.9) | 1102 (42.1) | 947 (36.2) | <0.0001 |
| Medical history, No. (%) |  |  |  |  |  |
| Ischemic stroke | 478 (18.3) | 507 (19.4) | 612 (23.4) | 640 (24.5) | <0.0001 |
| Diabetes | 583 (22.3) | 661 (25.3) | 611 (23.3) | 655 (25.0) | 0.03 |
| Hypertension | 1527 (58.3) | 1645 (62.8) | 1664 (63.6) | 1750 (66.8) | <0.0001 |
| Hypercholesterolemia | 227 (8.7) | 231 (8.8) | 207 (7.9) | 204 (7.8) | 0.42 |
| Coronary heart disease | 209 (8.0) | 251 (9.6) | 326 (12.5) | 349 (13.3) | <0.0001 |
| Atrial fibrillation | 99 (3.8) | 135 (5.2) | 202 (7.7) | 317 (12.1) | <0.0001 |
| Index event, No. (%) |  |  |  |  |  |
| Ischemic stroke | 2408 (92.0) | 2422 (92.5) | 2456 (93.8) | 2467 (94.2) | 0.003 |
| TIA | 210 (8.0) | 196 (7.5) | 162 (6.2) | 151 (5.8) | 0.003 |
| Baseline NIHSS, median (IQR) | 3 (1-5) | 3 (1-5) | 3 (1-6) | 4 (2-7) | <0.0001 |
| mRS score before the onset of index events≥2, No. (%) | 193 (7.4) | 204 (7.8) | 254 (9.7) | 269 (10.3) | 0.0002 |
| Leukocyte count (10^9^/L), median (IQR) | 6.9 (5.8-8.3) | 6.9 (5.7-8.4) | 6.8 (5.6-8.4) | 7.1 (5.8-8.6) | 0.0008 |
| LDL-C levels (mmol/L), median (IQR) | 2.3 (1.7-3.0) | 2.3 (1.8-3.0) | 2.3 (1.7-3.0) | 2.3 (1.7-3.0) | 0.45 |

Abbreviation: IQR = interquartile range; NIHSS=National Institutes of Health Stroke Scale; mRS=modified Rankin Scale; LDL-C, low-density lipoprotein cholesterol.

Additional Table 8. Median Marker Levels by Baseline Characteristics

|  | IL6, ng/L  Median (IQR) | IL-1Ra, ng/L  Median (IQR) | hsCRP, mg/L  Median (IQR) | Lp-PLA_2_, ug/L  Median (IQR) | Lp-PLA_2_-A, nmol/min/ml  Median (IQR) | YKL-40, mg/L  Median (IQR) |
| --- | --- | --- | --- | --- | --- | --- |
| Age (y) |  |  |  |  |  |  |
| <50 | 2.0 (1.3-3.6) | 329.3 (250.0-475.6) | 1.4 (0.7-3.5) | 168.8 (123.3-216.8) | 164.0 (128.4-194.9) | 37.7 (25.7-61.9) |
| 50-65 | 2.3 (1.5-4.3) | 331.8 (248.2-475.0) | 1.6 (0.8-4.0) | 175.5 (128.2-223.7) | 161.7 (127.9-193.2) | 56.1 (35.7-97.9) |
| 65-75 | 3.0 (1.8-5.6) | 350.2 (261.8-521.5) | 2.0 (0.9-5.4) | 177.3 (128.6-231.6) | 162.6 (126.6-198.0) | 82.1 (49.1-148.9) |
| ≥75 | 4.2 (2.4-8.6) | 366.7 (272.7-544.3) | 2.8 (1.1-8.8) | 174.0 (128.1-225.9) | 162.9 (129.7-193.8) | 122.6 (71.7-194.9) |
| Sex |  |  |  |  |  |  |
| Male | 2.6 (1.6-5.0) | 319.6 (242.6-450.5) | 1.7 (0.8-4.7) | 175.1 (127.1-225.4) | 166.9 (131.9-199.3) | 59.8 (36.3-108.9) |
| Femal | 2.7 (1.6-5.1) | 399.0 (293.0-591.3) | 1.9 (0.9-4.9) | 174.6 (128.3-224.4) | 152. 5 (120.6-182.9) | 78.6 (44.6-149.2) |
| Body mass index | |  |  |  |  |  |
| <24.9 | 2.7 (1.6-5.2) | 323.8 (242.2-466.7) | 1.6 (0.8-4.8) | 174.0 (125.2-225.1) | 160.9 (127.5-194.6) | 68.9 (39.6-131.7) |
| ≥24.9 | 2.6 (1.6-4.9) | 367.3 (274.4-533.9) | 2.0 (0.9-4.7) | 176.0 (130.8-225.2) | 163.9 (128.8-194.5) | 60.5 (36.9-113.0) |
| Smoking | |  |  |  |  |  |
| Yes | 2.7 (1.6-4.9) | 318.5 (241.1-450.9) | 1.7 (0.8-4.6) | 177.3 (129.4-227.7) | 167.7 (132.8-200.5) | 57.4 (35.5-105.6) |
| No | 2.6 (1.6-5.1) | 360.9 (267.5-536.0) | 1.8 (0.8-4.8) | 173.2 (126.2-222.9) | 158.6 (124.9-189.5) | 71.9 (41.3-137.3) |
| History of Ischemic stroke | |  |  |  |  |  |
| Yes | 2.9 (1.8-5.8) | 347.6 (258.0-499.3) | 2.0 (0.9-5.6) | 168.3 (123.6-220.4) | 157.5 (123.6-192.4) | 73.5 (41.7-138.0) |
| No | 2.6 (1.5-4.9) | 339.6 (253.7-496.8) | 1.7 (0.8-4.5) | 176.2 (128.9-226.1) | 163.5 (129.6-195.3) | 62.9 (37.4-119.4) |
| History of diabetes | |  |  |  |  |  |
| Yes | 2.8 (1.7-5.0) | 363.0 (273.1-539.6) | 2.0 (0.9-4.9) | 175.6 (123.7-227.0) | 159.5 (125.4-192.4) | 65.8 (39.3-127.7) |
| No | 2.6 (1.6-5.1) | 334.4 (249.9-485.9) | 1.7 (0.8-4.7) | 174.9 (128.7-224.6) | 163.3 (128.8-195.4) | 65.1 (37.8-121.7) |
| History of hypertension | |  |  |  |  |  |
| Yes | 2.7 (1.7-5.2) | 350.7 (262.7-517.4) | 1.9 (0.9-4.9) | 174.8 (127.3-225.2) | 161.9 (127.5-193.8) | 67.6 (39.6-129.3) |
| No | 2.5 (1.5-4.9) | 326.8 (242.2-462.0) | 1.6 (0.8-4.4) | 175.3 (128.0-225.0) | 163.2 (128.8-195.9) | 61.1 (36.1-112.6) |
| History of hypercholesterolemia | |  |  |  |  |  |
| Yes | 2.7 (1.7-4.6) | 358.3 (271.8-517.1) | 1.9 (0.9-4.6) | 179.0 (135.5-227.1) | 161.9 (130.2-193.9) | 61.2 (37.5-115.4) |
| No | 2.6 (1.6-5.1) | 340.0 (253.4-495.9) | 1.8 (0.8-4.8) | 174.8 (127.1-224.8) | 162.2 (127.9-194.7) | 65.6 (38.3-123.8) |
| History of coronary heart disease | |  |  |  |  |  |
| Yes | 3.3 (2.0-6.5) | 364.4 (272.7-539.2) | 2.5 (1.0-6.4) | 177.1 (130.9-225.2) | 162.1 (130.9-197.8) | 78.3 (45.7-142.9) |
| No | 2.6 (1.6-4.9) | 338.6 (252.6-493.1) | 1.7 (0.8-4.6) | 174.8 (127.2-225.1) | 162.3 (127.6-194.2) | 63.5 (37.5-120.2) |
| History of atrial fibrillation | |  |  |  |  |  |
| Yes | 5.1 (2.7-11.1) | 370.8 (270.5-566.3) | 3.6 (1.3-10.9) | 177.9 (129.3-230.8) | 163.9 (129.5-195.3) | 102.0 (55.2-186.9) |
| No | 2.5 (1.6-4.7) | 339.2 (253.4-492.8) | 1.7 (0.8-4.4) | 174.8 (127.4-224.6) | 162.0 (127.8-194.5) | 63.1 (37.5-117.9) |
| Index event |  |  |  |  |  |  |
| Ischemic | 2.7 (1.6-5.2) | 344.6 (256.4-500.6) | 1.8 (0.8-4.9) | 175.3 (127.4-225.9) | 162.8 (128.4-194.9) | 65.8 (38.4-124.3) |
| TIA | 1.9 (1.3-3.2) | 310.8 (235.6-427.1) | 1.2 (0.7-3.1) | 170.2 (128.9-216.3) | 154.9 (124.8-189.1) | 57.0 (35.0-107.1) |
| Baseline NIHSS | |  |  |  |  |  |
| ≤3 | 2.3 (1.4-4.1) | 324.3 (246.1-459.0) | 1.4 (0.8-3.5) | 173.1 (127.6-223.8) | 159.7 (127.4-192.4) | 60.9 (36.7-110.8) |
| 3-25 | 3.1 (1.8-6.3) | 365.0 (267.2-539.1) | 2.3 (1.0-6.2) | 176.4 (127.5-226.7) | 164.6 (128.8-197.1) | 70.5 (40.0-136.8) |
| >25 | 9.5 (5.7-15.5) | 365.8 (277.2-497.8) | 12.4 (4.0-19.6) | 194.9 (113.3-216.3) | 172.0 (142.4-189.4) | 90.2 (50.4-201.5) |
| mRS score before the onset of index events | | |  |  |  |  |
| 0-1 | 2.6 (1.6-4.9) | 339.0 (253.3-491.7) | 1.7 (0.8-4.6) | 175.3 (128.1-225.2) | 162.0 (128.4-194.5) | 64.4 (37.9-121.3) |
| ≥2 | 3.3 (1.9-6.9) | 372.5 (269.8-561.5) | 2.3 (0.9-6.6) | 169.5 (121.8-224.3) | 164.2 (124.4-195.6) | 74.5 (43.3-142.3) |
| Baseline leukocyte count | |  |  |  |  |  |
| <10*10^9^/L | 2.5 (1.6-4.7) | 334.8 (250.5-484.7) | 1.6 (0.8-4.3) | 175.3 (128.1-225.0) | 162.1 (127.9-194.3) | 64.8 (38.0-120.8) |
| ≥10*10^9^/L | 4.0 (2.1-9.7) | 395.0 (298.0-609.2) | 3.8 (1.4-12.2) | 170.4 (122.6-225.3) | 163.7 (129.3-195.9) | 68.4 (39.0-141.1) |

Abbreviation: IL-6=interleukin-6; IL-1Ra = interleukin-1 receptor antagonist; hsCRP= high sensitive C-reactive protein; Lp-PLA_2_= lipoprotein-associated phospolipase A_2_; Lp-PLA_2_-A= lipoprotein-associated phospolipase A2 activity; IQR = interquartile range; NIHSS=National Institutes of Health Stroke Scale; mRS=modified Rankin Scale.

Additional Table 9. The Correlation between Markers.

|  | IL-6 | IL-1Ra | hsCRP | Lp-PLA_2_ | Lp-PLA_2_-A | YKL-40 |
| --- | --- | --- | --- | --- | --- | --- |
| IL-6 | / | 0.21;<0.0001* | 0.45;<0.0001* | 0.01;0.25 | 0.03;0.006* | 0.29;<0.0001* |
| IL-1Ra | 0.21;<0.0001* | / | 0.24;<0.0001* | 0.02;0.02 | 0.02;0.02* | 0.15;<0.0001* |
| hsCRP | 0.45;<0.0001* | 0.24;<0.0001* | / | 0.005;0.61 | 0.008;0.41 | 0.22;<0.0001* |
| Lp-PLA_2_ | 0.01;0.25 | 0.02;0.02* | 0.005;0.61 | / | 0.74;<0.0001* | 0.04;<0.0001* |
| Lp-PLA_2_-A | 0.03;0.006* | 0.02;0.02* | 0.008;0.41 | 0.74;<0.0001* | / | -0.005;0.59 |
| YKL-40 | 0.29;<0.0001* | 0.15;<0.0001* | 0.22;<0.0001* | 0.04;<0.0001* | -0.005;0.59 | / |

Abbreviation: OR=odds ratio; HR = hazard ratio; CI=confidence intervals; IL-6=interleukin-6; IL-1Ra = interleukin-1 receptor antagonist; hsCRP= high sensitive C-reactive protein; Lp-PLA_2_= lipoprotein-associated phospolipase A_2_; Lp-PLA_2_-A= lipoprotein-associated phospolipase A_2_ activity. Spearman correlation analysis was performed and data were presented as r^2^.

Additional Table 10. Risk of Composite Vascular Events by Marker Levels within 1-Year.

| Marker and Levels * | | Events (No, (%)) | Model 1 † | | Model 2 ‡ | | Model 3 § | | Model 4 \| \| | |
| --- | --- | --- | --- | --- | --- | --- | --- | --- | --- | --- |
|  |  |  | HR (95% CI) | P Value | HR (95% CI) | P Value | HR (95% CI) | P Value | HR (95% CI) | P Value |
| IL-6  (ng/L) | Q1 | 218 (8.3) | Reference | / | Reference | / | Reference | / | Reference | / |
|  | Q2 | 223 (8.5) | 1.03 (0.85-1.24) | 0.79 | 0.94 (0.78-1.13) | 0.51 | 0.93 (0.77-1.13) | 0.47 | 0.92 (0.76-1.12) | 0.42 |
|  | Q3 | 279 (10.7) | 1.30 (1.09-1.55) | 0.004 | 1.10 (0.92-1.32) | 0.30 | 1.12 (0.93-1.35) | 0.22 | 1.11 (0.92-1.33) | 0.28 |
|  | Q4 | 364 (13.9) | 1.78 (1.50-2.10) | <0.0001 | 1.39 (1.16-1.66) | 0.0004 | 1.40 (1.17-1.69) | 0.0003 | 1.39 (1.16-1.67) | 0.0004 |
|  | Continuous model | | 1.35 (1.26-1.44) | <0.0001 | 1.23 (1.14-1.32) | <0.0001 | 1.23 (1.14-1.33) | <0.0001 | 1.23 (1.14-1.32) | <0.0001 |
| IL-1Ra  (ng/L) | Q1 | 254 (9.7) | Reference | / | Reference | / | Reference | / | Reference | / |
|  | Q2 | 255 (9.7) | 1.01 (0.85-1.20) | 0.92 | 0.97 (0.81-1.16) | 0.74 | 0.98 (0.82-1.17) | 0.79 | 0.98 (0.82-1.17) | 0.79 |
|  | Q3 | 298 (11.4) | 1.19 (1.01-1.41) | 0.04 | 1.09 (0.92-1.29) | 0.35 | 1.08 (0.91-1.29) | 0.38 | 1.08 (0.90-1.28) | 0.41 |
|  | Q4 | 277 (10.6) | 1.12 (0.95-1.33) | 0.19 | 0.99 (0.83-1.19) | 0.95 | 1.00 (0.84-1.21) | 0.96 | 0.99 (0.82-1.19) | 0.91 |
|  | Continuous model | | 1.11 (1.01-1.22) | 0.03 | 1.05 (0.95-1.16) | 0.31 | 1.05 (0.95-1.17) | 0.31 | 1.05 (0.95-1.16) | 0.38 |
| hsCRP  (mg/L) | Q1 | 219 (8.3) | Reference | / | Reference | / | Reference | / | Reference | / |
|  | Q2 | 225 (8.6) | 1.05 (0.87-1.26) | 0.63 | 1.03 (0.85-1.24) | 0.80 | 1.04 (0.86-1.26) | 0.70 | 1.03 (0.85-1.25) | 0.74 |
|  | Q3 | 284 (10.9) | 1.34 (1.12-1.59) | 0.001 | 1.24 (1.04-1.49) | 0.02 | 1.25 (1.04-1.50) | 0.02 | 1.23 (1.03-1.48) | 0.02 |
|  | Q4 | 356 (13.6) | 1.73 (1.46-2.05) | <0.0001 | 1.46 (1.22-1.74) | <0.0001 | 1.47 (1.23-1.76) | <0.0001 | 1.45 (1.21-1.74) | <0.0001 |
|  | Continuous model | | 1.17 (1.12-1.22) | <0.0001 | 1.11 (1.07-1.16) | <0.0001 | 1.12 (1.07-1.17) | <0.0001 | 1.12 (1.07-1.17) | <0.0001 |
| Lp-PLA_2_  (ug/L) | Q1 | 254 (9.7) | Reference | / | Reference | / | Reference | / | Reference | / |
|  | Q2 | 251 (9.6) | 1.00 (0.84-1.19) | 0.98 | 1.02 (0.86-1.22) | 0.81 | 1.01 (0.85-1.21) | 0.90 | 1.01 (0.84-1.20) | 0.93 |
|  | Q3 | 280 (10.7) | 1.12 (0.94-1.33) | 0.20 | 1.14 (0.96-1.36) | 0.12 | 1.07 (0.90-1.28) | 0.46 | 1.06 (0.89-1.27) | 0.52 |
|  | Q4 | 299 (11.4) | 1.20 (1.01-1.42) | 0.03 | 1.19 (1.01-1.41) | 0.04 | 1.12 (0.93-1.34) | 0.24 | 1.11 (0.92-1.33) | 0.28 |
|  | Continuous model | | 1.21 (1.05-1.39) | 0.008 | 1.20 (1.05-1.38) | 0.009 | 1.14 (0.98-1.33) | 0.09 | 1.13 (0.97-1.32) | 0.12 |
| Lp-PLA_2_-A  (nmol/min/ml) | Q1 | 241 (9.2) | Reference | / | Reference | / | Reference | / | Reference | / |
|  | Q2 | 245 (9.4) | 1.02 (0.86-1.22) | 0.81 | 1.02 (0.86-1.22) | 0.80 | 1.00 (0.84-1.20) | 0.98 | 1.00 (0.83-1.19) | 0.96 |
|  | Q3 | 298 (11.4) | 1.26 (1.06-1.49) | 0.008 | 1.25 (1.05-1.48) | 0.01 | 1.19 (1.00-1.43) | 0.05 | 1.19 (0.99-1.42) | 0.06 |
|  | Q4 | 300 (11.5) | 1.28 (1.08-1.51) | 0.005 | 1.28 (1.08-1.52) | 0.005 | 1.22 (1.01-1.46) | 0.04 | 1.21 (1.00-1.45) | 0.05 |
|  | Continuous model | | 1.33 (1.11-1.59) | 0.002 | 1.33 (1.11-1.60) | 0.002 | 1.25 (1.02-1.52) | 0.03 | 1.23 (1.01-1.50) | 0.04 |
| YKL-40  (mg/L) | Q1 | 225 (8.6) | Reference | / | Reference | / | Reference | / | Reference | / |
|  | Q2 | 249 (9.5) | 1.11 (0.93-1.33) | 0.25 | 1.06 (0.88-1.27) | 0.56 | 1.07 (0.89-1.29) | 0.50 | 1.06 (0.88-1.27) | 0.57 |
|  | Q3 | 266 (10.2) | 1.20 (1.00-1.43) | 0.05 | 1.05 (0.87-1.27) | 0.59 | 1.07 (0.88-1.29) | 0.51 | 1.06 (0.87-1.28) | 0.58 |
|  | Q4 | 344 (13.1) | 1.59 (1.34-1.88) | <0.0001 | 1.30 (1.08-1.56) | 0.006 | 1.33 (1.10-1.61) | 0.003 | 1.32 (1.09-1.59) | 0.004 |
|  | Continuous model | | 1.28 (1.18-1.38) | <0.0001 | 1.15 (1.05-1.26) | 0.003 | 1.17 (1.06-1.28) | 0.001 | 1.16 (1.06-1.27) | 0.002 |
| IL-6+YKL-40 | Q1 | 257 (8.0) | Reference | / | Reference | / | Reference | / | Reference | / |
|  | Q2 | 184 (9.0) | 1.13 (0.94-1.37) | 0.21 | 1.04 (0.86-1.27) | 0.67 | 1.07 (0.88-1.31) | 0.50 | 1.07 (0.87-1.30) | 0.53 |
|  | Q3 | 217 (10.6) | 1.35 (1.13-1.62) | 0.001 | 1.21 (1.01-1.45) | 0.04 | 1.25 (1.03-1.50) | 0.02 | 1.24 (1.03-1.49) | 0.03 |
|  | Q4 | 426 (13.3) | 1.74 (1.49-2.03) | <0.0001 | 1.37 (1.16-1.63) | 0.0003 | 1.41 (1.19-1.68) | 0.0001 | 1.40 (1.17-1.66) | 0.0002 |

Abbreviation: HR = hazard ratio; CI=confidence intervals; Q1=quartile 1; Q2=quartile 2; Q3=quartile 3; Q4=quartile 4; IL-6=interleukin-6; IL-6R= interleukin-6 receptor; IL-1Ra = interleukin-1 receptor antagonist; hsCRP= high sensitive C-reactive protein; Lp-PLA_2_= lipoprotein-associated phospolipase A_2_; Lp-PLA_2_-A= lipoprotein-associated phospolipase A_2_ activity.

* All markers were categorized into 4 even groups by quartiles. In the continuous model, the hazards ratios correspond to per-unit increment of logarithm of marker value.

† Model 1: unadjusted;

‡ Model 2: adjusted for age, sex, body mass index, smoking, index event, medical histories of atrial fibrillation, coronary heart disease, ischemic stroke, diabetes, hypertension and hypercholesterolemia, baseline NIHSS score and baseline leukocyte count.

§ Model 3: adjusted for all factors in model 2 and baseline low-density lipoprotein cholesterol levels;

| | Model 4: adjusted for all factors in model 3 and usage of antiplatelet, antihypertensive, hypoglycemic and statin during 1-year follow-up period.

Additional Table 11. Associations of IL-6 and YKL-40 with recurrent stroke within one year according to TOAST (Trial of ORG 10172 in Acute Stroke Treatment) classification.

| Subtype | Marker and Levels * | | Events (No, (%)) | Model 1 † | | Model 2 ‡ | | Model 3 § | |
| --- | --- | --- | --- | --- | --- | --- | --- | --- | --- |
|  |  |  |  | HR (95% CI) | P Value | HR (95% CI) | P Value | HR (95% CI) | P Value |
| Large-artery Atherosclerosis  n=2637 | IL-6  (ng/L) | Q1 | 51 (10.8) | Reference | / | Reference | / | Reference | / |
|  |  | Q2 | 65 (10.9) | 1.01 (0.70-1.46) | 0.97 | 0.94 (0.65-1.36) | 0.75 | 0.92 (0.63-1.34) | 0.66 |
|  |  | Q3 | 79 (10.8) | 1.00 (0.70-1.42) | 0.99 | 0.87 (0.61-1.25) | 0.46 | 0.89 (0.62-1.28) | 0.52 |
|  |  | Q4 | 141 (16.9) | 1.66 (1.21-2.29) | 0.002 | 1.29 (0.91-1.83) | 0.15 | 1.28 (0.90-1.82) | 0.17 |
|  | YKL-40  (mg/L) | Q1 | 59 (9.8) | Reference | / | Reference | / | Reference | / |
|  |  | Q2 | 86 (12.7) | 1.31 (0.94-1.83) | 0.11 | 1.28 (0.91-1.78) | 0.15 | 1.19 (0.85-1.67) | 0.32 |
|  |  | Q3 | 80 (11.5) | 1.18 (0.85-1.66) | 0.33 | 1.05 (0.74-1.49) | 0.79 | 1.01 (0.71-1.43) | 0.97 |
|  |  | Q4 | 111 (16.8) | 1.81 (1.32-2.48) | 0.0002 | 1.50 (1.07-2.12) | 0.02 | 1.46 (1.03-2.06) | 0.03 |
| Cardioembolism  n=679 | IL-6  (ng/L) | Q1 | 10 (11.9) | Reference | / | Reference | / | Reference | / |
|  |  | Q2 | 16 (11.7) | 1.00 (0.45-2.19) | 0.99 | 0.94 (0.42-2.12) | 0.89 | 1.00 (0.44-2.25) | 1.00 |
|  |  | Q3 | 22 (12.2) | 1.04 (0.49-2.19) | 0.92 | 0.95 (0.43-2.06) | 0.89 | 0.97 (0.44-2.12) | 0.93 |
|  |  | Q4 | 36 (13.0) | 1.15 (0.57-2.32) | 0.69 | 1.00 (0.47-2.15) | 0.99 | 1.05 (0.49-2.25) | 0.90 |
|  | YKL-40  (mg/L) | Q1 | 10 (9.3) | Reference | / | Reference | / | Reference | / |
|  |  | Q2 | 16 (12.5) | 1.35 (0.61-2.97) | 0.46 | 1.32 (0.59-2.97) | 0.50 | 1.38 (0.61-3.13) | 0.44 |
|  |  | Q3 | 22 (12.4) | 1.37 (0.65-2.89) | 0.41 | 1.27 (0.59-2.77) | 0.54 | 1.27 (0.58-2.78) | 0.55 |
|  |  | Q4 | 36 (13.6) | 1.53 (0.76-3.09) | 0.23 | 1.41 (0.66-3.02) | 0.38 | 1.32 (0.61-2.82) | 0.48 |
| Small-vessel Occlusion  n=2178 | IL-6  (ng/L) | Q1 | 37 (5.7) | Reference | / | Reference | / | Reference | / |
|  |  | Q2 | 45 (7.1) | 1.25 (0.81-1.93) | 0.31 | 1.12 (0.72-1.75) | 0.62 | 1.08 (0.68-1.71) | 0.74 |
|  |  | Q3 | 36 (7.1) | 1.24 (0.78-1.96) | 0.36 | 1.11 (0.69-1.78) | 0.67 | 1.10 (0.68-1.81) | 0.69 |
|  |  | Q4 | 36 (9.2) | 1.67 (1.06-2.64) | 0.03 | 1.36 (0.83-2.21) | 0.22 | 1.31 (0.79-2.19) | 0.30 |
|  | YKL-40  (mg/L) | Q1 | 34 (5.5) | Reference | / | Reference | / | Reference | / |
|  |  | Q2 | 41 (6.7) | 1.24 (0.79-1.96) | 0.35 | 1.22 (0.77-1.94) | 0.40 | 1.38 (0.85-2.24) | 0.19 |
|  |  | Q3 | 36 (7.1) | 1.32 (0.82-2.10) | 0.25 | 1.23 (0.75-2.01) | 0.42 | 1.25 (0.74-2.12) | 0.41 |
|  |  | Q4 | 43 (9.7) | 1.84 (1.17-2.88) | 0.008 | 1.68 (1.02-2.75) | 0.04 | 1.89 (1.12-3.18) | 0.02 |
| Stroke of Other Determined Etiology  n=118 | IL-6  (ng/L) | Q1 | 3 (7.7) | Reference | / | Reference | / | Reference | / |
|  |  | Q2 | 1 (5.9) | 0.76 (0.08-7.31) | 0.81 | 0.62 (0.05-7.07) | 0.70 | 0.44 (0.03-5.99) | 0.54 |
|  |  | Q3 | 4 (12.9) | 1.70 (0.38-7.59) | 0.49 | 1.57 (0.27-9.09) | 0.62 | 1.41 (0.25-8.09) | 0.70 |
|  |  | Q4 | 4 (12.9) | 1.79 (0.40-7.99) | 0.45 | 0.68 (0.11-4.27) | 0.68 | 0.57 (0.07-4.38) | 0.59 |
|  | YKL-40  (mg/L) | Q1 | 3 (9.4) | Reference | / | Reference | / | Reference | / |
|  |  | Q2 | 1 (3.9) | 0.41 (0.04-3.91) | 0.44 | 0.27 (0.02-3.11) | 0.29 | 0.24 (0.02-2.97) | 0.27 |
|  |  | Q3 | 3 (12.0) | 1.28 (0.26-6.33) | 0.76 | 1.49 (0.24-9.47) | 0.67 | 1.24 (0.19-8.36) | 0.82 |
|  |  | Q4 | 5 (14.3) | 1.65 (0.39-6.89) | 0.50 | 1.12 (0.18-6.94) | 0.90 | 0.88 (0.13-6.08) | 0.90 |
| Stroke of Undetermined Etiology  n=4860 | IL-6  (ng/L) | Q1 | 112 (8.1) | Reference | / | Reference | / | Reference | / |
|  |  | Q2 | 85 (6.9) | 0.85 (0.64-1.12) | 0.24 | 0.78 (0.59-1.04) | 0.09 | 0.77 (0.58-1.03) | 0.08 |
|  |  | Q3 | 121 (10.4) | 1.30 (1.00-1.68) | 0.05 | 1.10 (0.85-1.44) | 0.47 | 1.10 (0.84-1.44) | 0.48 |
|  |  | Q4 | 122 (11.3) | 1.46 (1.13-1.89) | 0.004 | 1.16 (0.88-1.54) | 0.28 | 1.16 (0.87-1.54) | 0.32 |
|  | YKL-40  (mg/L) | Q1 | 111 (8.9) | Reference | / | Reference | / | Reference | / |
|  |  | Q2 | 94 (8.0) | 0.90 (0.68-1.18) | 0.44 | 0.85 (0.64-1.12) | 0.25 | 0.86 (0.65-115) | 0.32 |
|  |  | Q3 | 112 (9.2) | 1.05 (0.81-1.36) | 0.73 | 0.93 (0.70-1.22) | 0.59 | 0.94 (0.71-1.25) | 0.69 |
|  |  | Q4 | 123 (10.1) | 1.17 (0.91-1.51) | 0.23 | 0.95 (0.71-1.25) | 0.69 | 0.97 (0.73-1.30) | 0.84 |

Abbreviation: HR = hazard ratio; CI=confidence intervals; Q1=quartile 1; Q2=quartile 2; Q3=quartile 3; Q4=quartile 4; IL-6=interleukin-6; IL-1Ra = interleukin-1 receptor antagonist; hsCRP= high sensitive C-reactive protein; Lp-PLA_2_= lipoprotein-associated phospolipase A_2_; Lp-PLA_2_-A= lipoprotein-associated phospolipase A_2_ activity.

* All markers were categorized into 4 even groups by quartiles.

† Model 1: unadjusted;

‡ Model 2: adjusted for age, sex, body mass index, smoking, index event, medical histories of atrial fibrillation, coronary heart disease, ischemic stroke, diabetes, hypertension and hypercholesterolemia, baseline NIHSS score and baseline leukocyte count.

§ Model 3: adjusted for all factors in model 2 and baseline low-density lipoprotein cholesterol levels and usage of antiplatelet, antihypertensive, hypoglycemic and statin during 1-year follow-up period.

Additional Table 12. Associations of individual inflammation marker or combination of IL-6 and YKL-40 with an Modified Rankin Scale score ≥3 within one year.

| Marker and Levels * | | Events (No, (%)) | Model 1 † | | Model 2 ‡ | | Model 3 § | | Model 4 \| \| | |
| --- | --- | --- | --- | --- | --- | --- | --- | --- | --- | --- |
|  |  |  | OR (95% CI) | P Value | OR (95% CI) | P Value | OR (95% CI) | P Value | OR (95% CI) | P Value |
| IL-6  (ng/L) | Q1 | 150 (5.8) | Reference | / | Reference | / | Reference | / | Reference | / |
|  | Q2 | 225 (8.8) | 1.56 (1.26-1.93) | <0.0001 | 1.21 (0.97-1.52) | 0.09 | 1.23 (0.98-1.54) | 0.07 | 1.26 (1.00-1.59) | 0.05 |
|  | Q3 | 330 (12.9) | 2.40 (1.96-2.93) | <0.0001 | 1.43 (1.16-1.78) | 0.001 | 1.46 (1.18-1.81) | 0.0006 | 1.46 (1.17-1.83) | 0.0009 |
|  | Q4 | 648 (25.7) | 5.60 (4.64-6.75) | <0.0001 | 2.29 (1.86-2.83) | <0.0001 | 2.36 (1.91-2.91) | <0.0001 | 2.33 (1.87-2.90) | <0.0001 |
|  | Continuous model | | 2.19 (2.05-2.34) | <0.0001 | 1.51 (1.40-1.63) | <0.0001 | 1.53 (1.41-1.65) | <0.0001 | 1.50 (1.38-1.63) | <0.0001 |
| IL-1Ra  (ng/L) | Q1 | 236 (9.2) | Reference | / | Reference | / | Reference | / | Reference | / |
|  | Q2 | 261 (10.2) | 1.12 (0.93-1.34) | 0.24 | 0.99 (0.81-1.21) | 0.94 | 1.00 (0.81-1.22) | 0.97 | 0.98 (0.80-1.21) | 0.86 |
|  | Q3 | 361 (14.1) | 1.61 (1.36-1.92) | <0.0001 | 1.27 (1.05-1.54) | 0.02 | 1.27 (1.05-1.55) | 0.01 | 1.27 (1.03-1.55) | 0.02 |
|  | Q4 | 495 (19.6) | 2.40 (2.03-2.83) | <0.0001 | 1.67 (1.38-2.02) | <0.0001 | 1.65 (1.37-2.00) | <0.0001 | 1.74 (1.42-2.12) | <0.0001 |
|  | Continuous model | | 1.62 (1.49-1.76) | <0.0001 | 1.39 (1.26-1.53) | <0.0001 | 1.38 (1.24-1.52) | <0.0001 | 1.41 (1.27-1.57) | <0.0001 |
| hsCRP  (mg/L) | Q1 | 197 (7.6) | Reference | / | Reference | / | Reference | / | Reference | / |
|  | Q2 | 241 (9.5) | 1.27 (1.05-1.55) | 0.02 | 1.16 (0.94-1.43) | 0.16 | 1.16 (0.94-1.43) | 0.17 | 1.17 (0.94-1.45) | 0.16 |
|  | Q3 | 319 (12.5) | 1.74 (1.44-2.09) | <0.0001 | 1.35 (1.11-1.65) | 0.003 | 1.37 (1.12-1.67) | 0.002 | 1.32 (1.07-1.62) | 0.01 |
|  | Q4 | 596 (23.6) | 3.75 (3.16-4.45) | <0.0001 | 1.83 (1.51-2.21) | <0.0001 | 1.86 (1.53-2.25) | <0.0001 | 1.79 (1.47-2.18) | <0.0001 |
|  | Continuous model | | 1.49 (1.44-1.55) | <0.0001 | 1.21 (1.16-1.27) | <0.0001 | 1.22 (1.17-1.28) | <0.0001 | 1.21 (1.15-1.27) | <0.0001 |
| Lp-PLA_2_  (ug/L) | Q1 | 325 (12.7) | Reference | / | Reference | / | Reference | / | Reference | / |
|  | Q2 | 320 (12.6) | 0.99 (0.84-1.17) | 0.88 | 0.99 (0.82-1.19) | 0.89 | 0.99 (0.82-1.19) | 0.90 | 0.97 (0.80-1.18) | 0.77 |
|  | Q3 | 336 (13.1) | 1.04 (0.88-1.22) | 0.68 | 1.06 (0.88-1.27) | 0.55 | 1.05 (0.87-1.26) | 0.61 | 1.01 (0.84-1.22) | 0.93 |
|  | Q4 | 372 (14.6) | 1.18 (1.00-1.38) | 0.05 | 1.10 (0.92-1.31) | 0.32 | 1.09 (0.91-1.31) | 0.34 | 1.05 (0.87-1.26) | 0.64 |
|  | Continuous model | | 1.11 (0.97-1.26) | 0.13 | 1.06 (0.92-1.22) | 0.44 | 1.06 (0.91-1.22) | 0.46 | 1.02 (0.88-1.18) | 0.83 |
| Lp-PLA_2_-A  (nmol/min/ml) | Q1 | 329 (12.9) | Reference | / | Reference | / | Reference | / | Reference | / |
|  | Q2 | 315 (12.3) | 0.95 (0.81-1.12) | 0.54 | 0.99 (0.82-1.18) | 0.87 | 0.99 (0.82-1.19) | 0.91 | 0.98 (0.81-1.18) | 0.81 |
|  | Q3 | 345 (13.5) | 1.06 (0.90-1.24) | 0.51 | 0.99 (0.83-1.19) | 0.94 | 0.99 (0.83-1.19) | 0.95 | 0.95 (0.79-1.15) | 0.61 |
|  | Q4 | 364 (14.3) | 1.13 (0.97-1.33) | 0.13 | 1.09 (0.91-1.30) | 0.36 | 1.09 (0.91-1.30) | 0.37 | 1.03 (0.85-1.24) | 0.80 |
|  | Continuous model | | 1.15 (0.97-1.36) | 0.11 | 1.09 (0.90-1.31) | 0.38 | 1.09 (0.90-1.31) | 0.39 | 1.02 (0.84-1.24) | 0.85 |
| YKL-40  (mg/L) | Q1 | 201 (7.9) | Reference | / | Reference | / | Reference | / | Reference | / |
|  | Q2 | 258 (10.1) | 1.31 (1.08-1.58) | 0.007 | 1.06 (0.86-1.30) | 0.61 | 1.06 (0.86-1.31) | 0.57 | 1.06 (0.85-1.32) | 061 |
|  | Q3 | 333 (13.0) | 1.75 (1.46-2.10) | <0.0001 | 1.08 (0.88-1.33) | 0.45 | 1.09 (0.89-1.34) | 0.40 | 1.09 (0.88-1.35) | 0.42 |
|  | Q4 | 561 (22.1) | 3.31 (2.79-3.93) | <0.0001 | 1.57 (1.28-1.91) | <0.0001 | 1.61 (1.32-1.97) | <0.0001 | 1.59 (1.29-1.96) | <0.0001 |
|  | Continuous model | | 1.89 (1.74-2.05) | <0.0001 | 1.26 (1.14-1.38) | <0.0001 | 1.28 (1.16-1.41) | <0.0001 | 1.26 (1.14-1.39) | <0.0001 |
| IL-6+YKL-40 | Q1 | 185 (5.9) | Reference | / | Reference | / | Reference | / | Reference | / |
|  | Q2 | 190 (9.5) | 1.68 (1.36-2.07) | <0.0001 | 1.19 (0.95-1.49) | 0.12 | 1.21 (0.96-1.51) | 0.10 | 1.22 (0.96-1.53) | 0.10 |
|  | Q3 | 274 (13.9) | 2.57 (2.12-3.13) | <0.0001 | 1.60 (1.30-1.98) | <0.0001 | 1.62 (1.31-2.00) | <0.0001 | 1.60 (1.29-1.99) | <0.0001 |
|  | Q4 | 704 (22.7) | 4.69 (3.96-5.56) | <0.0001 | 1.91 (1.58-2.32) | <0.0001 | 1.96 (1.62-2.38) | <0.0001 | 1.91 (1.56-2.34) | <0.0001 |

Abbreviation: OR = odds ratio; CI=confidence intervals; Q1=quartile 1; Q2=quartile 2; Q3=quartile 3; Q4=quartile 4; IL-6=interleukin-6; IL-1Ra = interleukin-1 receptor antagonist; hsCRP= high sensitive C-reactive protein; Lp-PLA_2_= lipoprotein-associated phospolipase A_2_; Lp-PLA_2_-A= lipoprotein-associated phospolipase A_2_ activity.

* All markers were categorized into 4 even groups by quartiles. In the continuous model, the odds ratios correspond to per-unit increment of logarithm of marker value.

† Model 1: unadjusted.

‡ Model 2: adjusted for age, sex, body mass index, smoking, medical histories of atrial fibrillation, coronary heart disease, ischemic stroke, diabetes, hypertension and hypercholesterolemia, baseline NIHSS score, mRS score before the onset of index events and baseline leukocyte count.

§ Model 3: adjusted for all factors in Model 2 and tPA treatment.

| | Model 4: adjusted for all factors in Model 3 and stroke recurrence within 1 year.

Additional Figure 1. Flow Chart Showing the Participant Selection. Abbreviation: IL-6=interleukin-6; IL-1Ra = interleukin-1 receptor antagonist; hsCRP= high sensitive C-reactive protein; Lp-PLA_2_= lipoprotein-associated phospolipase A_2_; Lp-PLA_2_-A= lipoprotein-associated phospolipase A_2_ activity.


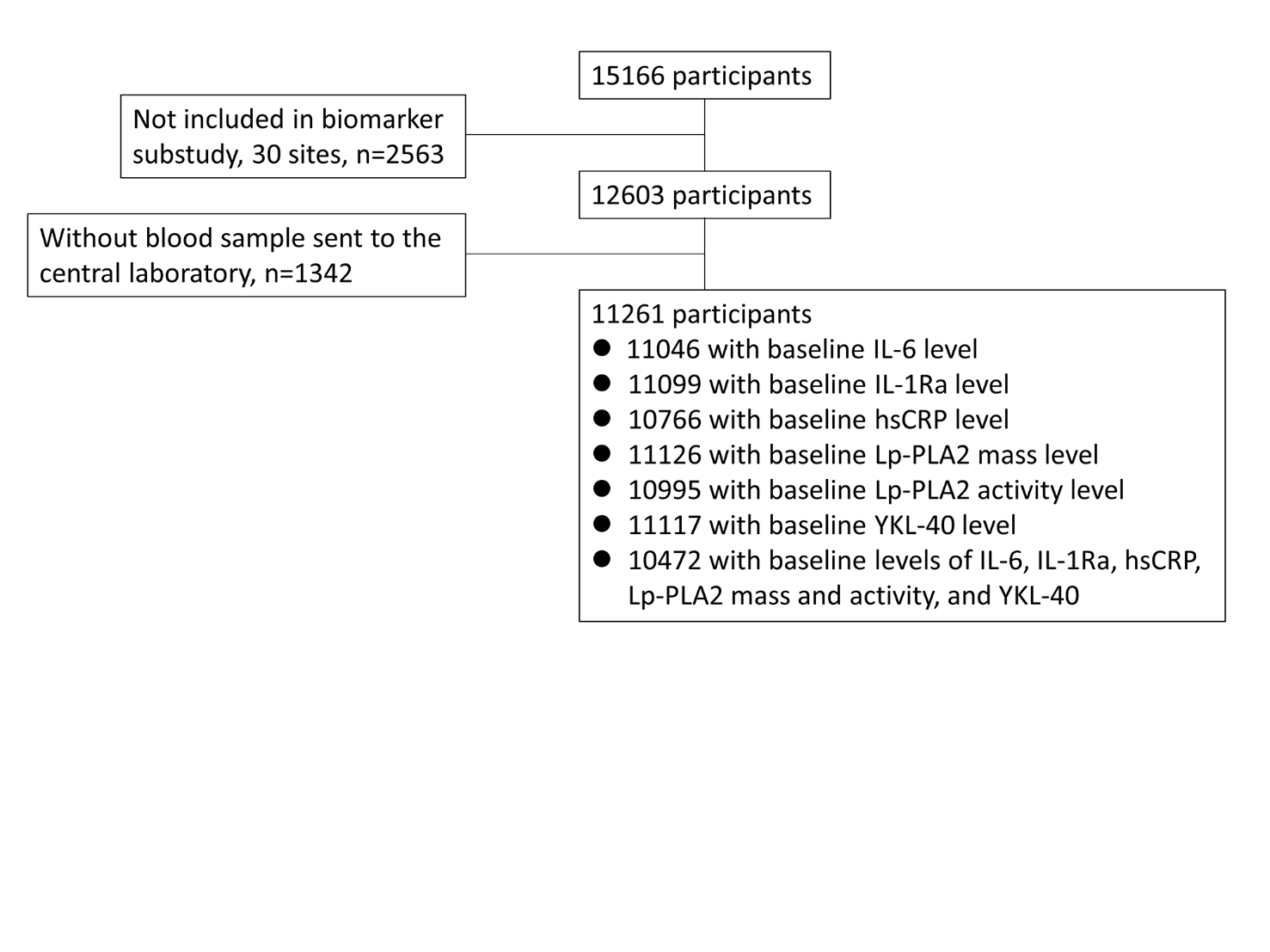


Additional Figure 2. Kaplan-Meier curves showing the probability of survival free of stroke during 1-year follow-up according to quartiles of levels of IL-6 (A), IL-1Ra (B), hsCRP (C), Lp-PLA_2_ (D), Lp-PLA_2_-A (E), YKL-40 (F). The inset shows the same data on an enlarged segment of the y axis. Abbreviation: IL-6=interleukin-6; IL-1Ra = interleukin-1 receptor antagonist; hsCRP= high sensitive C-reactive protein; Lp-PLA_2_= lipoprotein-associated phospolipase A_2_; Lp-PLA_2_-A= lipoprotein-associated phospolipase A_2_ activity.


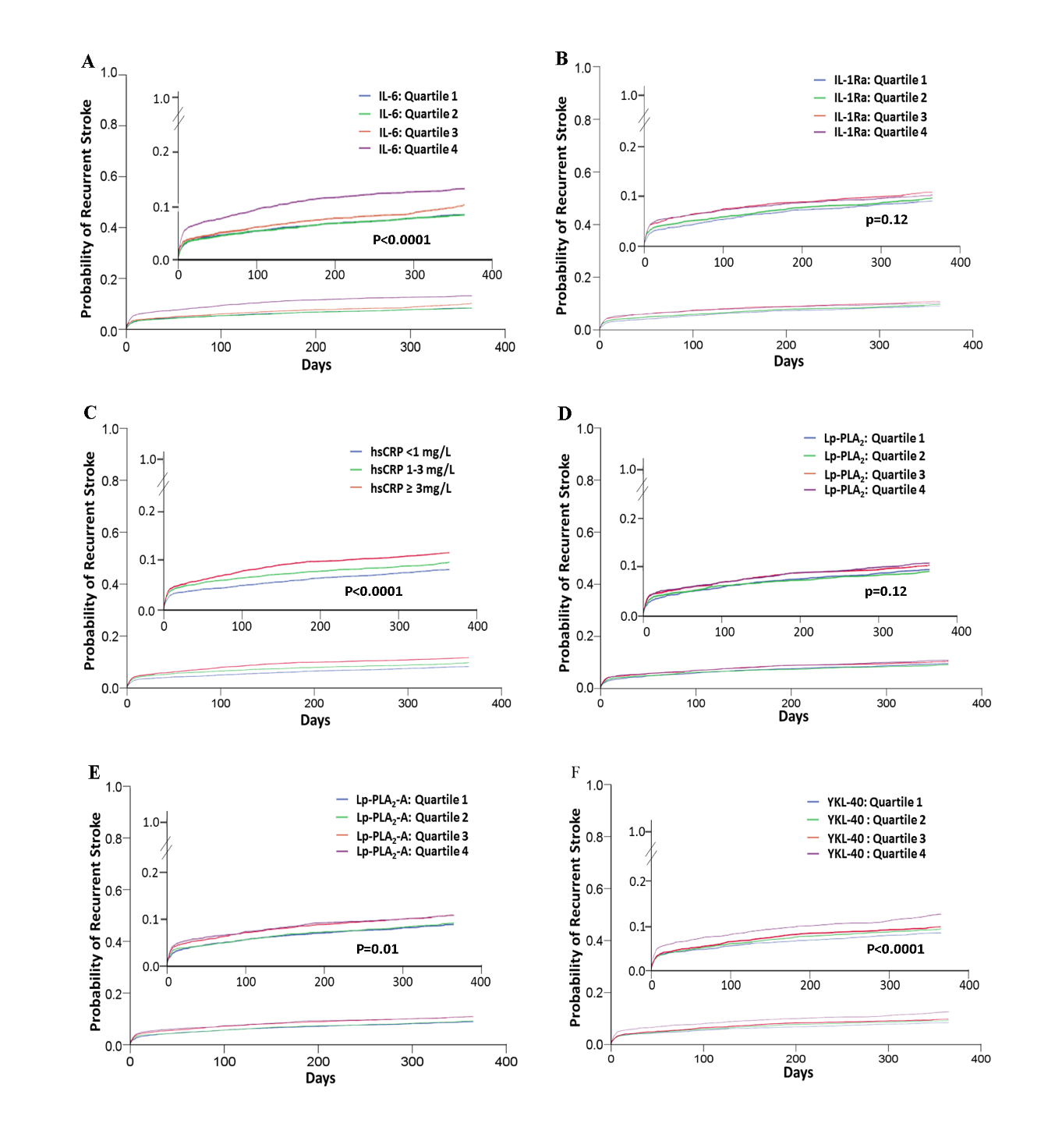


Additional Figure 3. Continuous adjusted hazard ratios for recurrent stroke according to inflammation markers of (A) IL-6, (B) IL-1Ra, (C) hsCRP, (D) Lp-PLA_2_, (E) Lp-PLA_2_-A and (F) YKL-40. Red line indicates adjusted hazard ratio, and blue lines indicate the 95% confidence interval bands. Covariates in the model include age, sex, body mass index, smoking, index event, medical histories of atrial fibrillation, coronary heart disease, ischemic stroke, diabetes, hypertension and hypercholesterolemia, baseline NIHSS score, and baseline leukocyte count, low-density lipoprotein cholesterol levels and usage of antiplatelet, antihypertensive, hypoglycemic and statin during 1-year follow-up period. Abbreviation: IL-6=interleukin-6; IL-1Ra = interleukin-1 receptor antagonist; hsCRP= high sensitive C-reactive protein; Lp-PLA_2_= lipoprotein-associated phospolipase A_2_; Lp-PLA_2_-A= lipoprotein-associated phospolipase A_2_ activity.


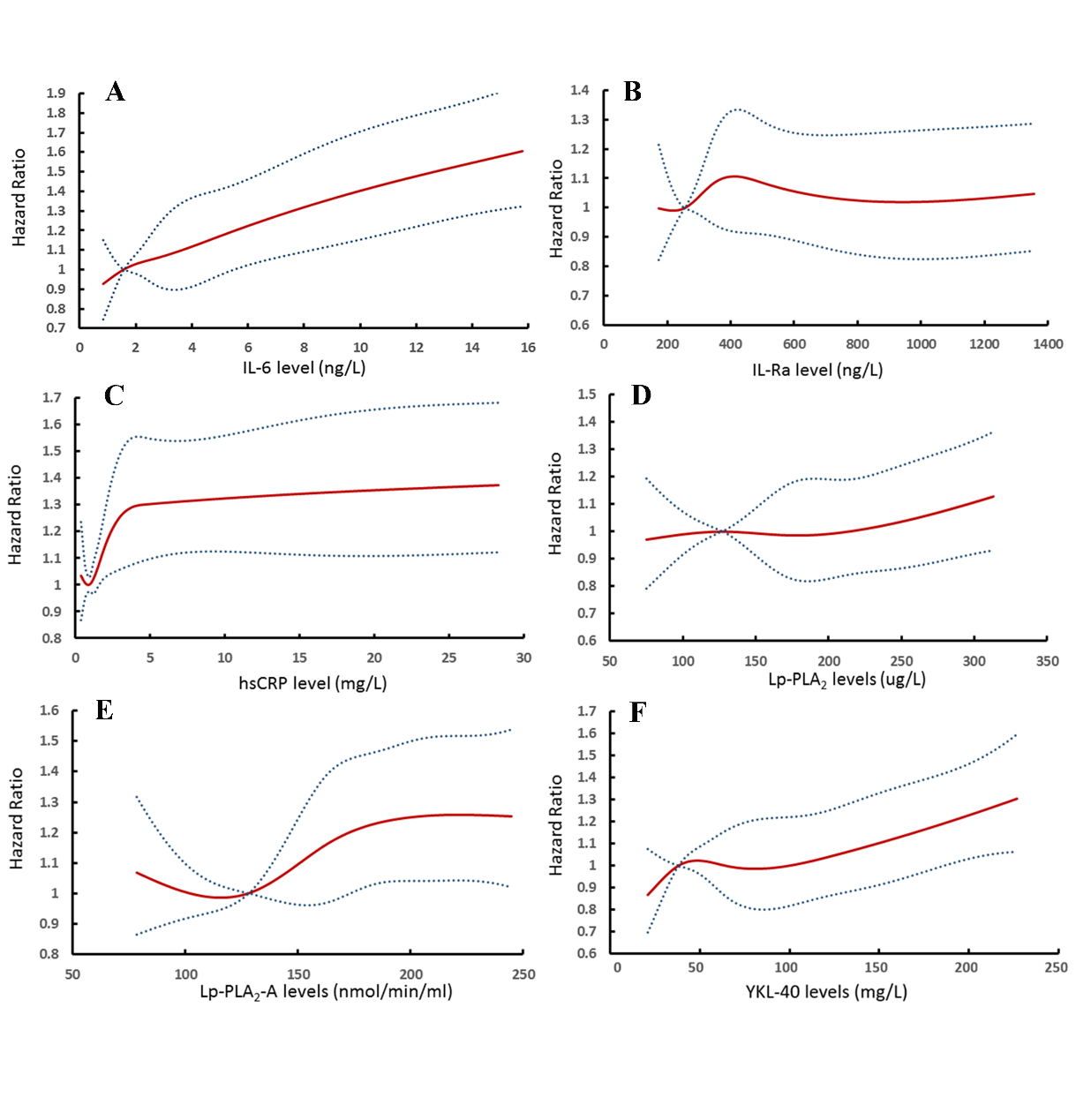


Additional Figure 4. Predictors of stroke recurrence within 1 year in the multivariate stepwise logistic regression analysis. All inflammatory markers were included in the model in addition to variables of age, sex, body mass index, smoking, index event, medical histories of atrial fibrillation, coronary heart disease, ischemic stroke, diabetes, hypertension and hypercholesterolemia, baseline NIHSS score, and baseline leukocyte count and low-density lipoprotein cholesterol levels. The odds ratios of inflammatory markers correspond to per-unit increment of marker value. Abbreviation: CI = confidence interval; IL-6=interleukin-6; TIA= transient ischemic attack; mRS=modified Rankin Scale; NIHSS= National Institutes of Health Stroke Scale.


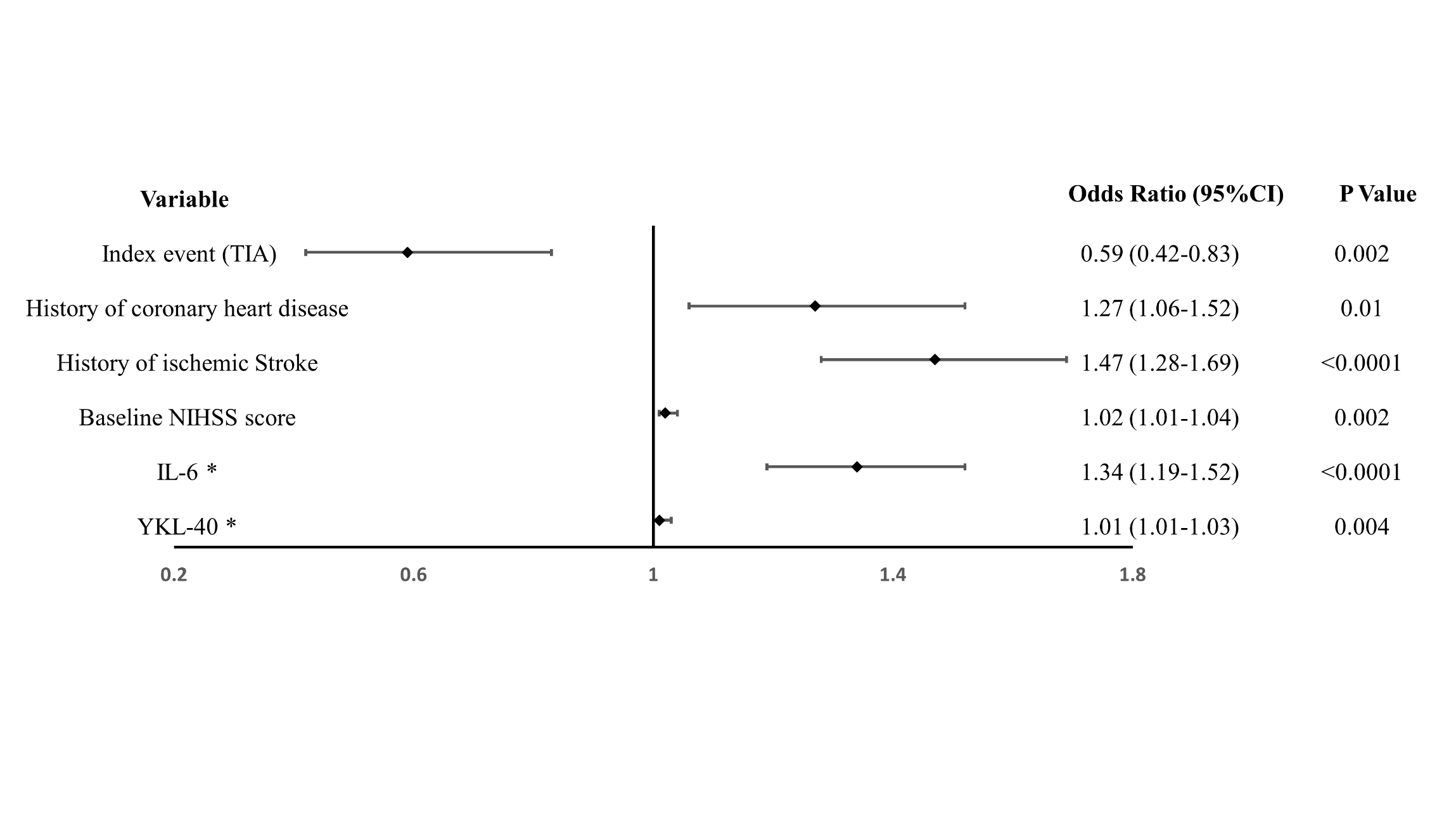


Additional Figure 5. Modified Rankin Scale Scores stratified according to quartiles of (A) IL-6, (B) IL-1Ra, (C) hsCRP, (D) Lp-PLA_2_, (E) Lp-PLA_2_-A and (F) YKL-40. Scores range from 0 to 6, with 0 indicating no symptoms, 1 no clinically significant disability, 2 slight disability, 3 moderate disability, 4 moderately severe disability, 5 severe disability and 6 death. In the multivariate regression model, variables include age, sex, body mass index, current or previous smoking, medical histories of atrial fibrillation, coronary heart disease, ischemic stroke, diabetes, hypertension and hypercholesterolemia, baseline NIHSS score, modified Rankin scale score before the onset of index events, baseline leukocyte count, intravenous recombinant tissue plasminogen activator (alteplase) treatment and stroke recurrence within 1 year. All markers or marker scores were categorized into 4 even groups by quartiles. Abbreviation: mRS= modified Rankin scale ; OR = odds ratio; CI = confidence interval; Q1=quartile 1; Q2=quartile 2; Q3=quartile 3; Q4=quartile 4; IL-6=interleukin-6; IL-1Ra = interleukin-1 receptor antagonist; hsCRP= high sensitive C-reactive protein; Lp-PLA_2_= lipoprotein-associated phospolipase A_2_; Lp-PLA_2_-A= lipoprotein-associated phospolipase A_2_ activity.


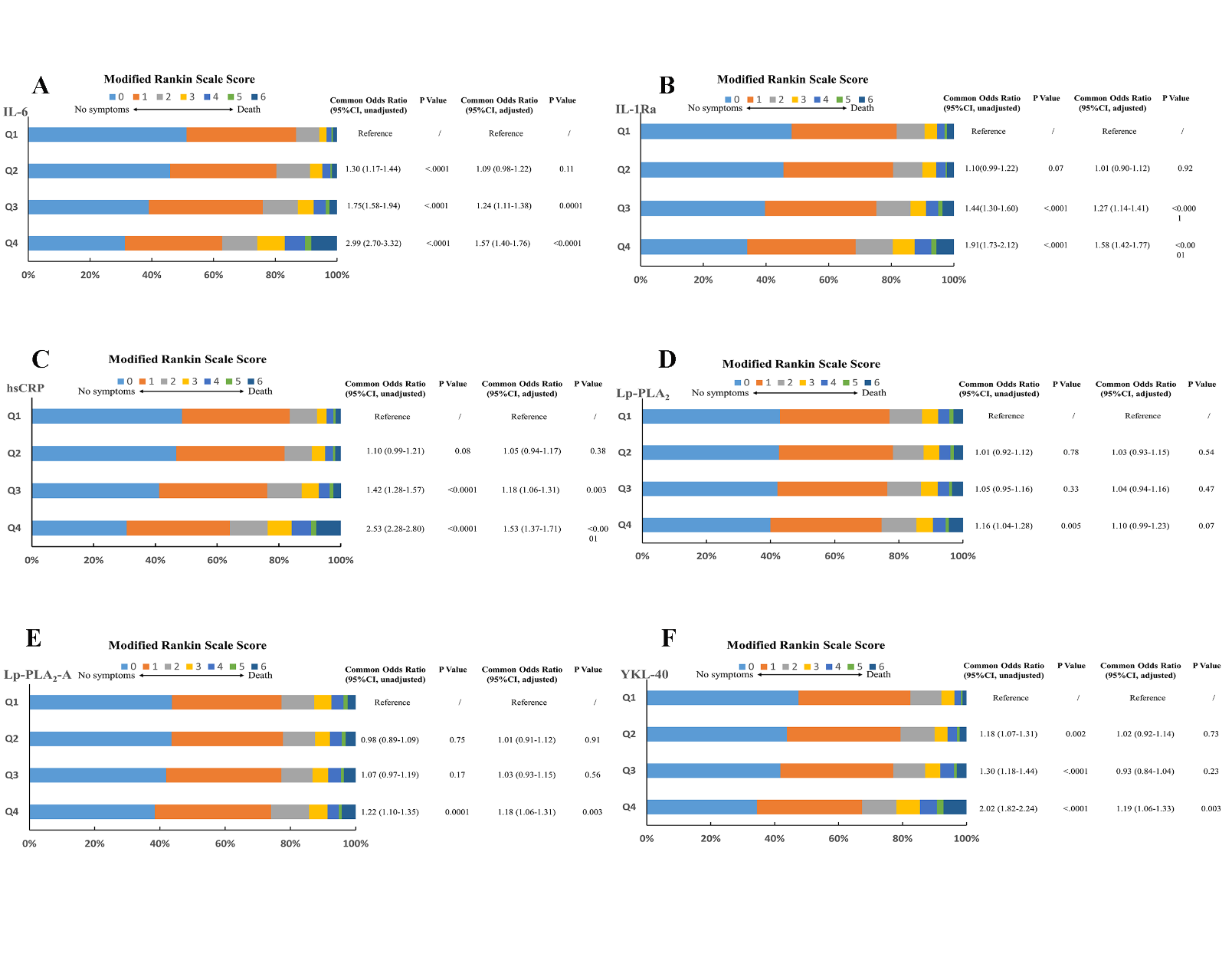


Additional Figure 6. Predictors of an Modified Rankin Scale score ≥2 within 1-year in the multivariate stepwise logistic regression analysis. All inflammatory markers were included in the model in addition to variables of age, sex, body mass index, current or previous smoking, index event, medical histories of atrial fibrillation, coronary heart disease, ischemic stroke, diabetes, hypertension and hypercholesterolemia, baseline NIHSS score, modified Rankin scale score before the onset of index events, baseline leukocyte count, intravenous recombinant tissue plasminogen activator (alteplase) treatment, and stroke recurrence within 1 year. The odds ratios of inflammatory markers correspond to per-unit increment of marker value. The inset shows the same data on an enlarged segment of the x axis for variables of baseline NIHSS, mRS before and YKL-40. Abbreviation: CI = confidence interval; TIA= transient ischemic attack; NIHSS= National Institutes of Health Stroke Scale; mRS=modified Rankin Scale; rtPA= recombinant tissue plasminogen activator; IL-6=interleukin-6.


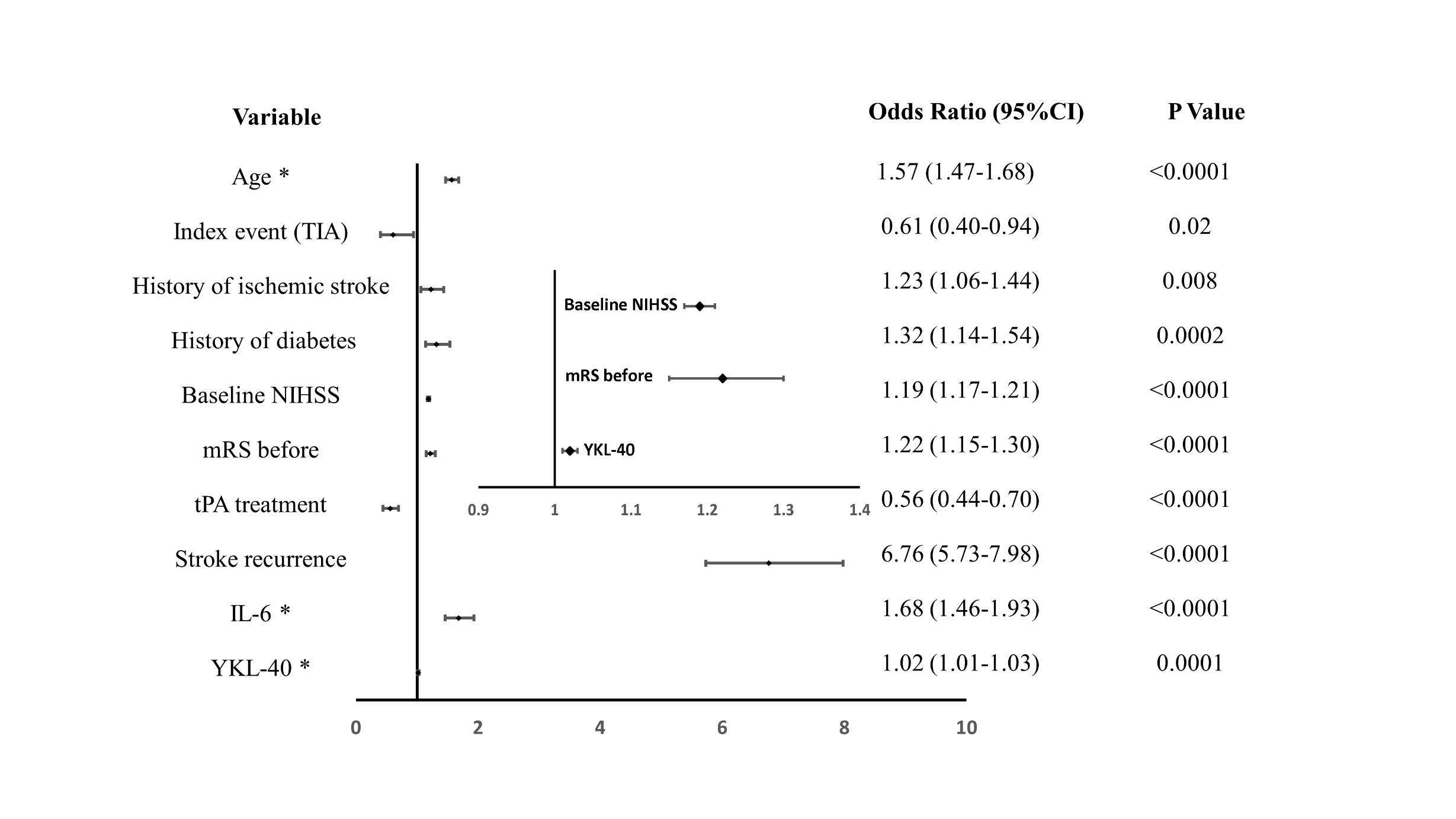

Supplement: Supplementary file 1 — Additional file 1. Supplemental tables and figures on participant selection, baseline characteristics and the associations of markers with outcomes. [file 12974_2022_2467_MOESM1_ESM.docx]
